# Supplementary figures and images for: Type II and IV toxin-antitoxin systems coordinately stabilize the integrative and conjugative element of the ICESa2603 family conferring multiple drug resistance in Streptococcus suis
Source: PLoS Pathog. 2024 Apr 19;20(4):e1012169. doi: 10.1371/journal.ppat.1012169 (PMC11062541; doi:10.1371/journal.ppat.1012169)

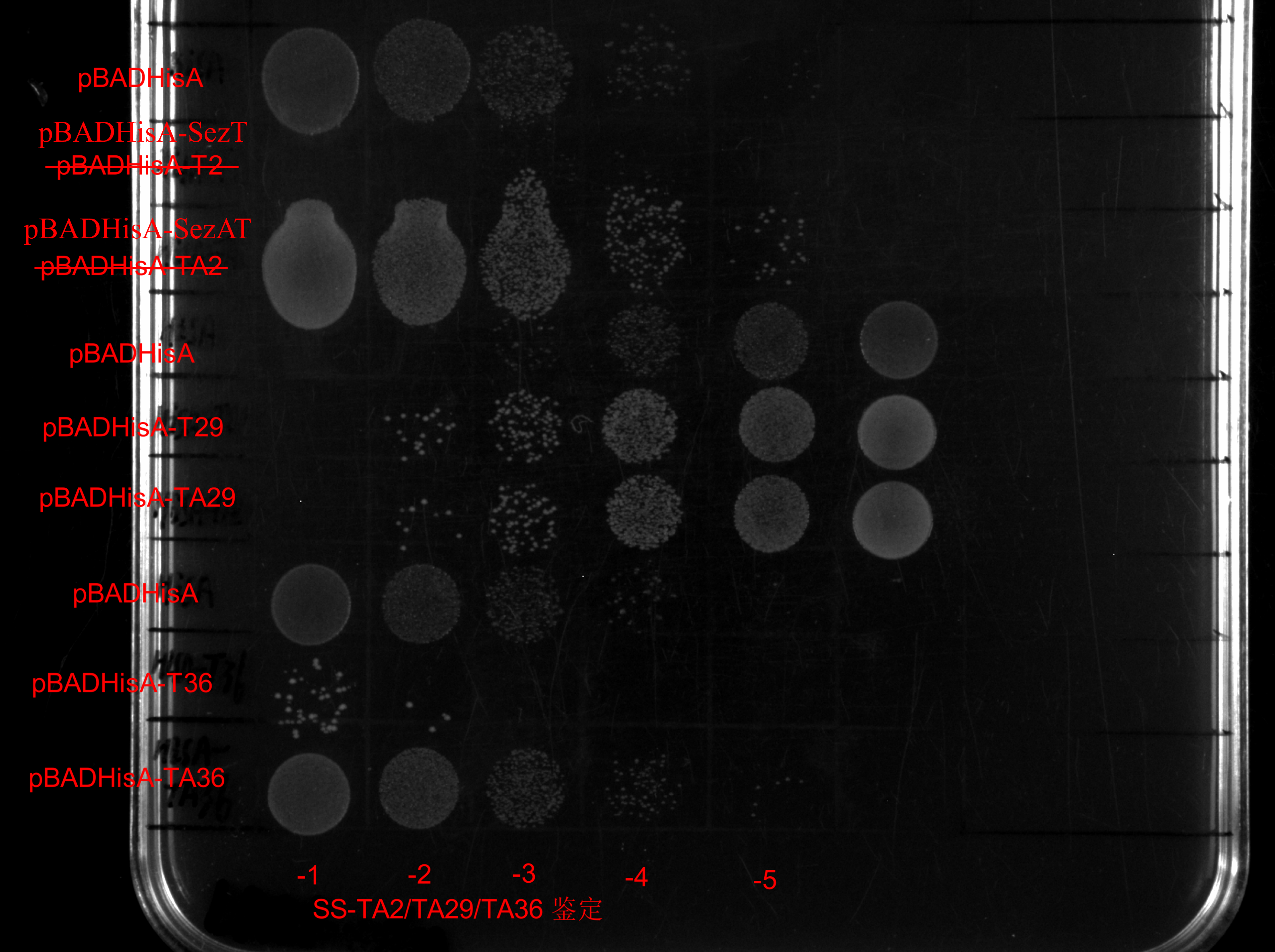

Supplement: S2 Data — Two text files containing all the amino acid sequences for Fig 6E and 6F. (ZIP) [file ppat.1012169.s002.zip › S2_Data/Figure 1B.tif]

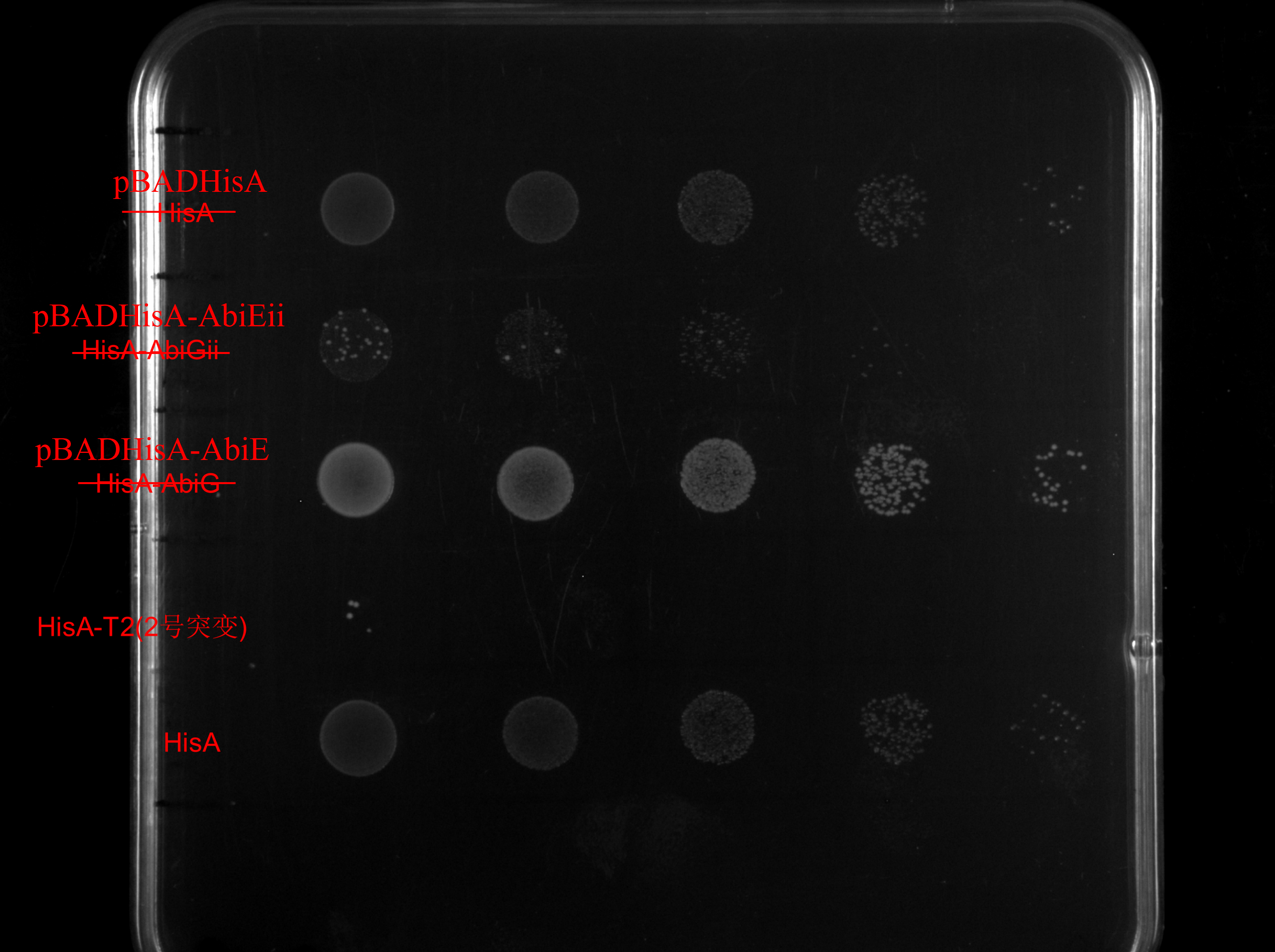

Supplement: S2 Data — Two text files containing all the amino acid sequences for Fig 6E and 6F. (ZIP) [file ppat.1012169.s002.zip › S2_Data/Figure 1D.tif]

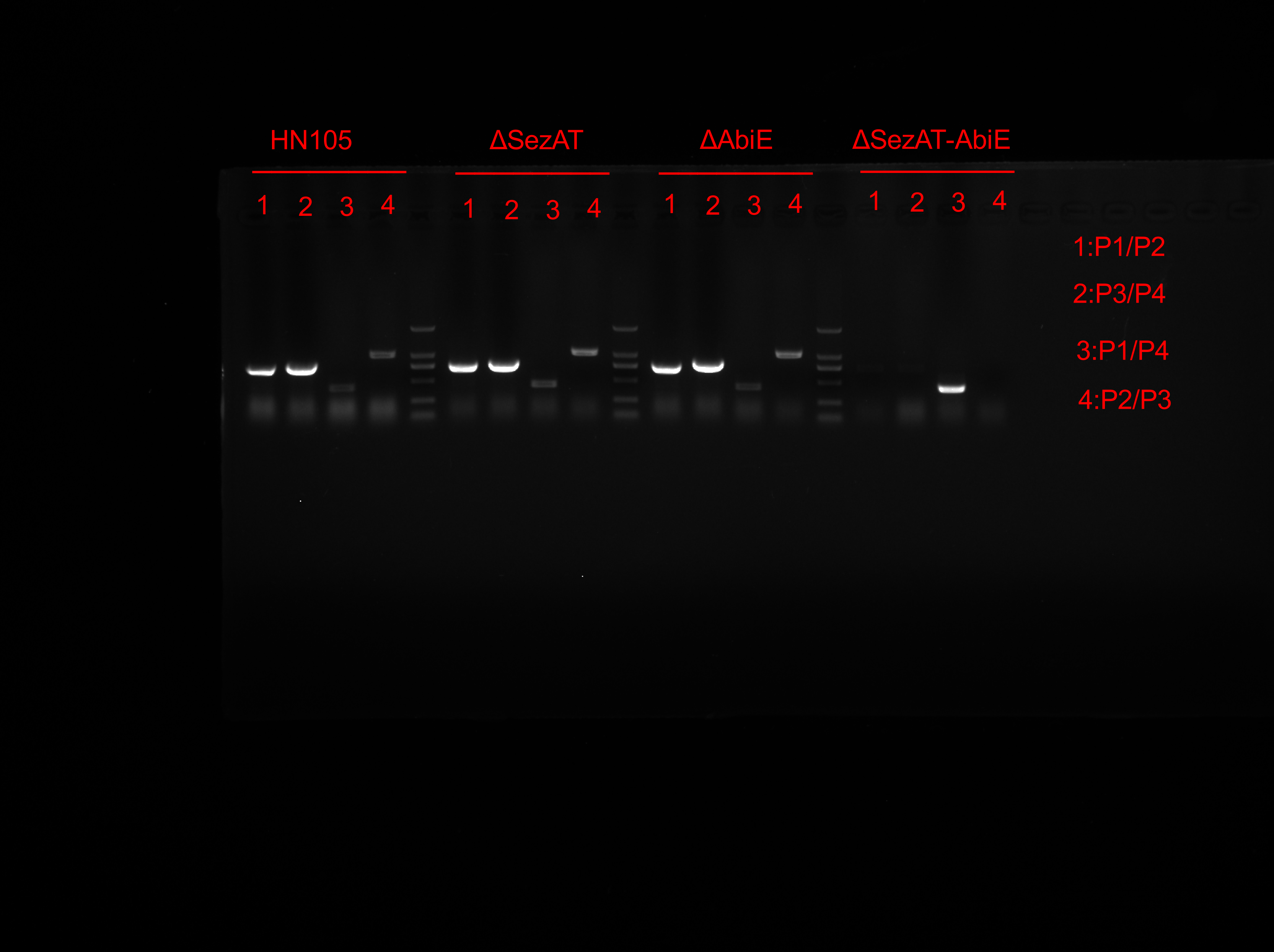

Supplement: S2 Data — Two text files containing all the amino acid sequences for Fig 6E and 6F. (ZIP) [file ppat.1012169.s002.zip › S2_Data/Figure 2B.tif]

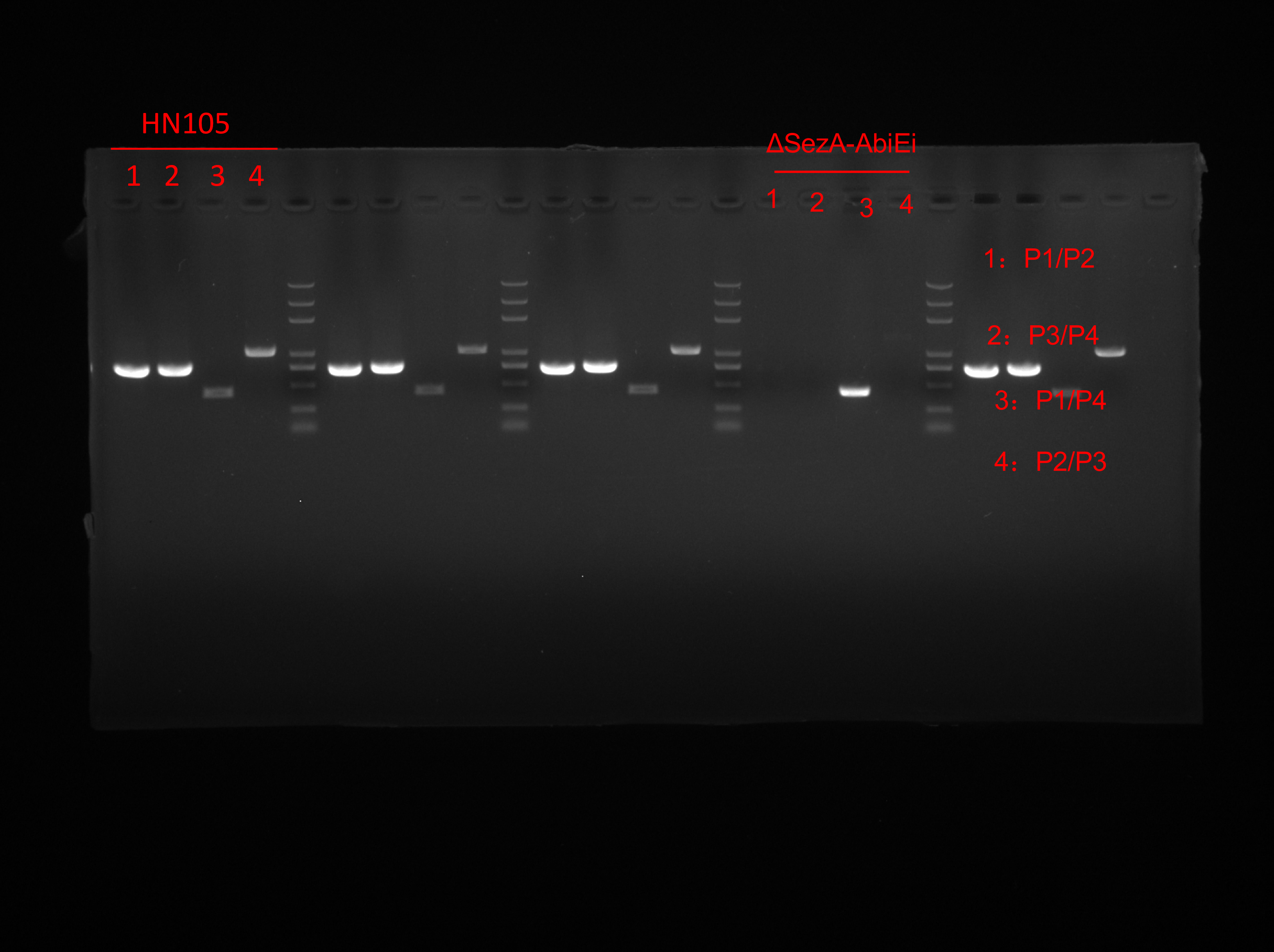

Supplement: S2 Data — Two text files containing all the amino acid sequences for Fig 6E and 6F. (ZIP) [file ppat.1012169.s002.zip › S2_Data/Figure 2E (Double deletion of SezA and AbiEi).tif]

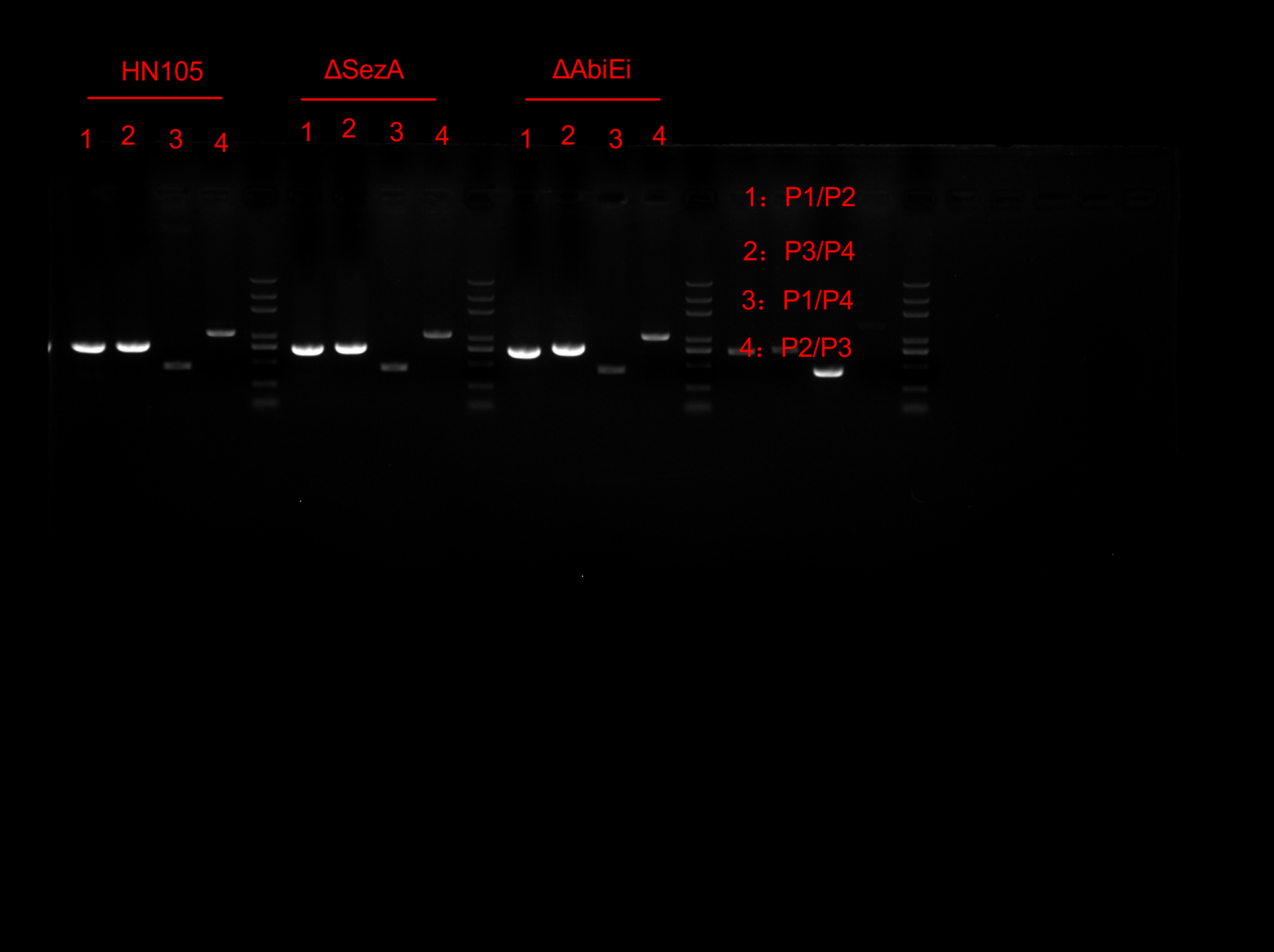

Supplement: S2 Data — Two text files containing all the amino acid sequences for Fig 6E and 6F. (ZIP) [file ppat.1012169.s002.zip › S2_Data/Figure 2E (HN105íóSingle deletion of SezA and AbiEi).tif]

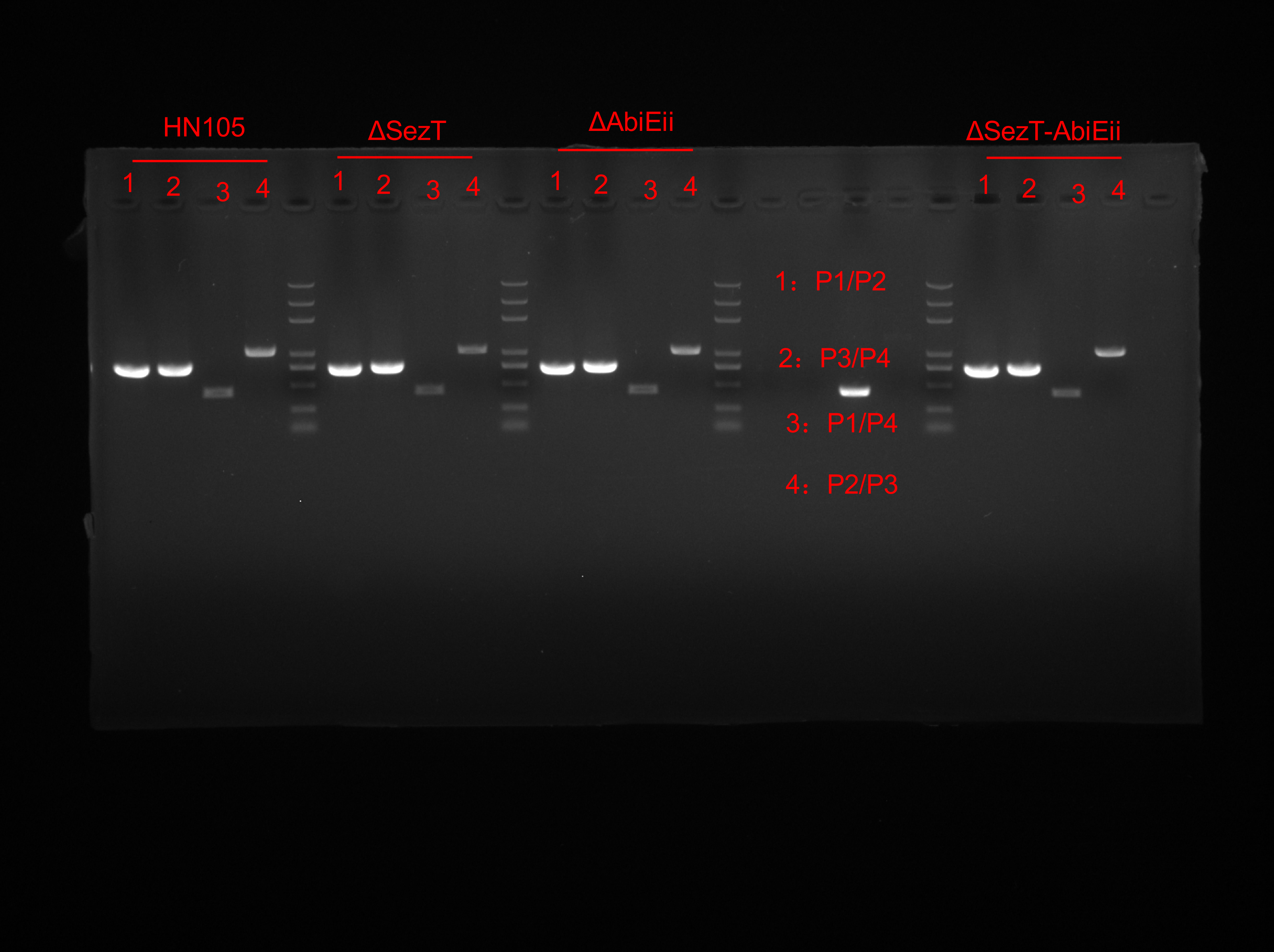

Supplement: S2 Data — Two text files containing all the amino acid sequences for Fig 6E and 6F. (ZIP) [file ppat.1012169.s002.zip › S2_Data/Figure 2F.tif]

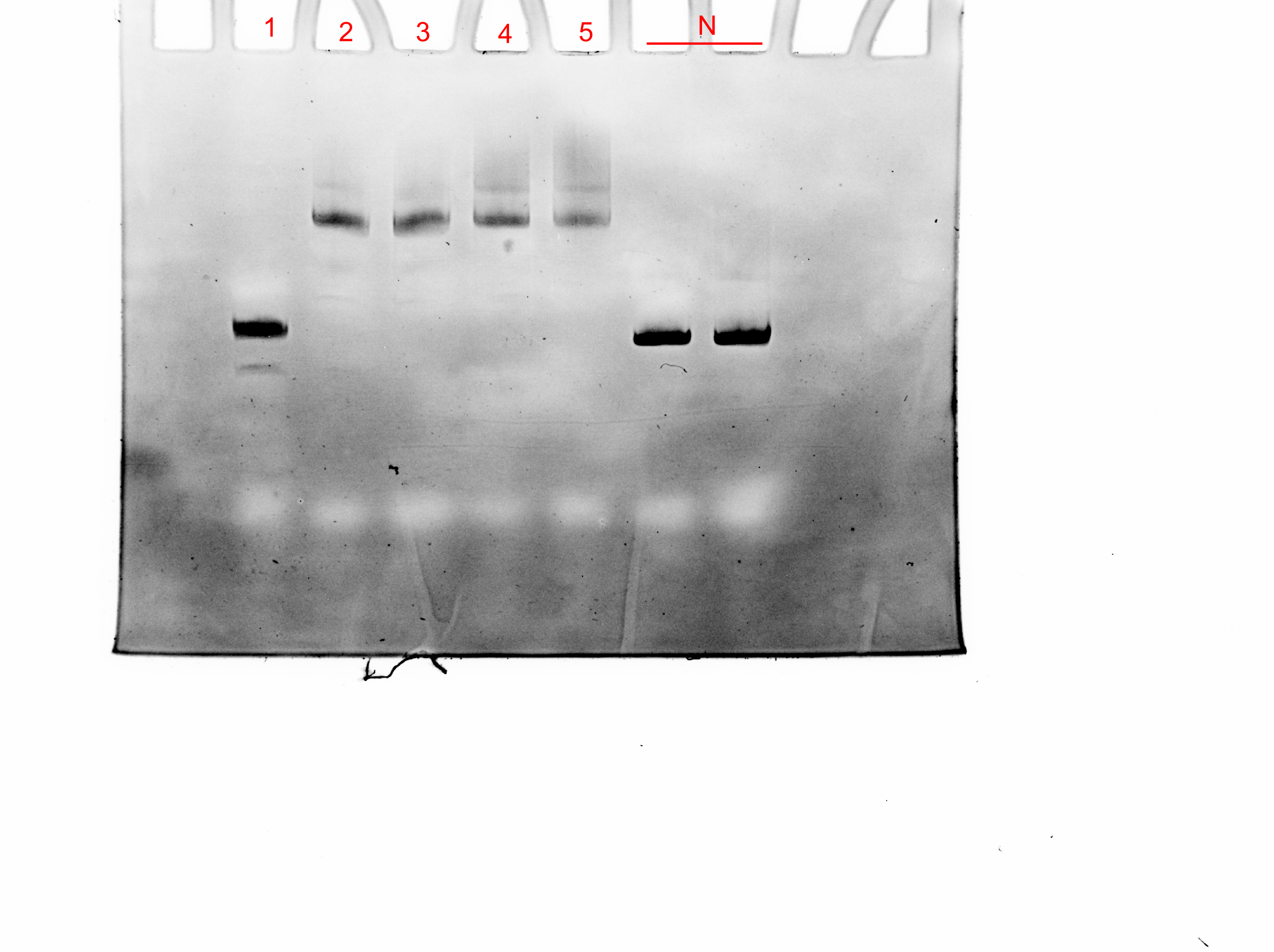

Supplement: S2 Data — Two text files containing all the amino acid sequences for Fig 6E and 6F. (ZIP) [file ppat.1012169.s002.zip › S2_Data/Figure 3B.tif]

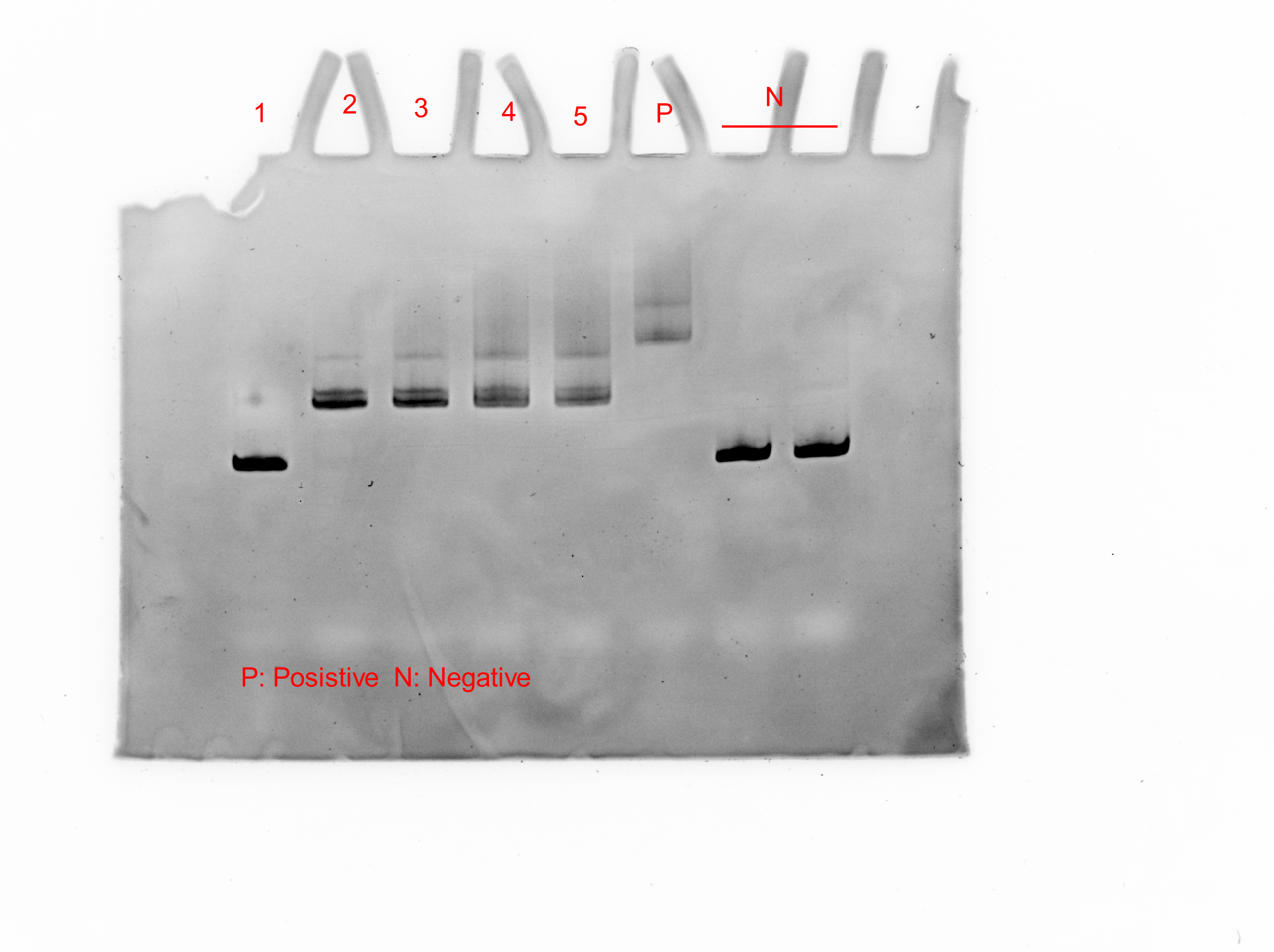

Supplement: S2 Data — Two text files containing all the amino acid sequences for Fig 6E and 6F. (ZIP) [file ppat.1012169.s002.zip › S2_Data/Figure 3C.tif]

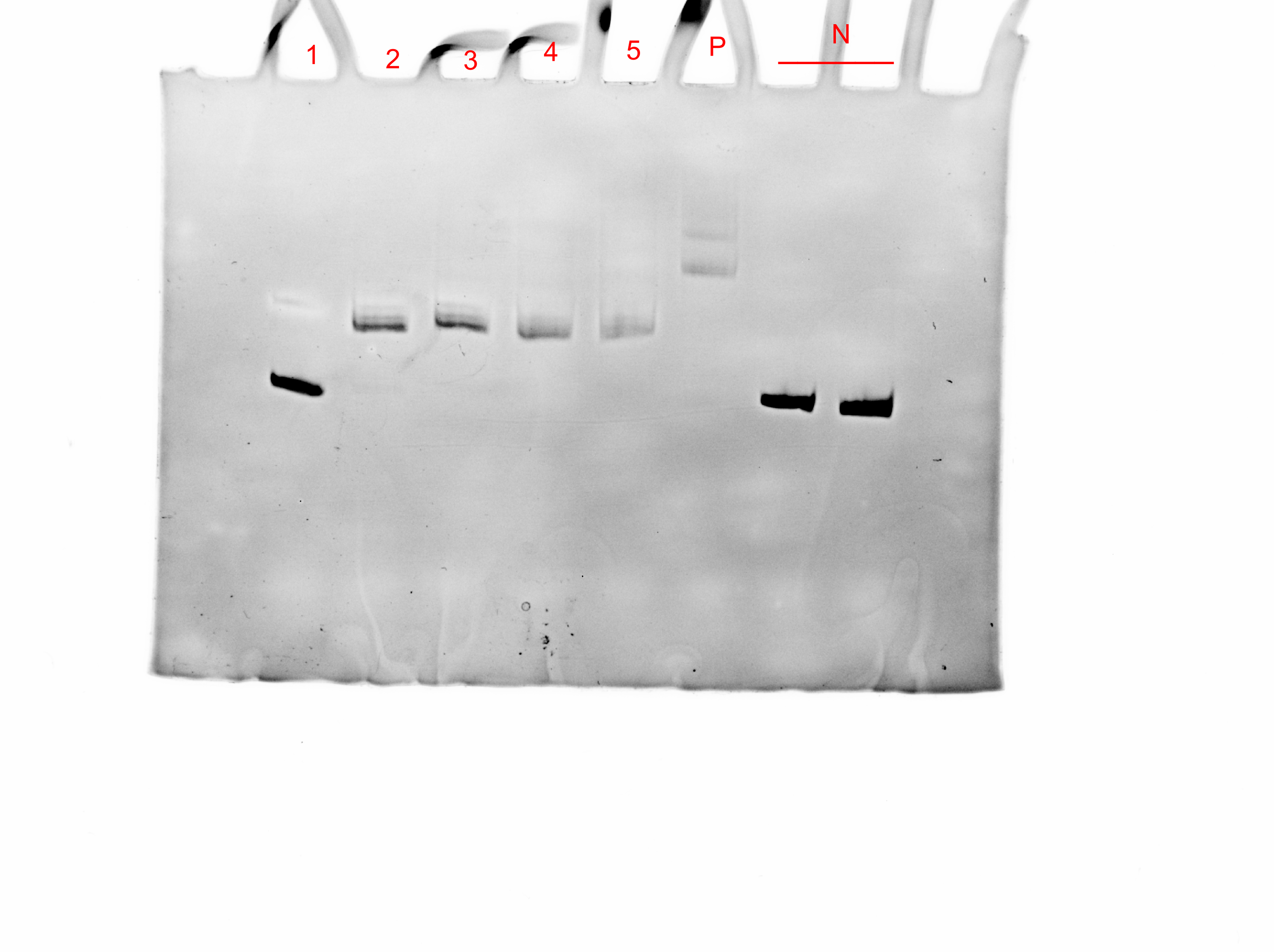

Supplement: S2 Data — Two text files containing all the amino acid sequences for Fig 6E and 6F. (ZIP) [file ppat.1012169.s002.zip › S2_Data/Figure 3D.tif]

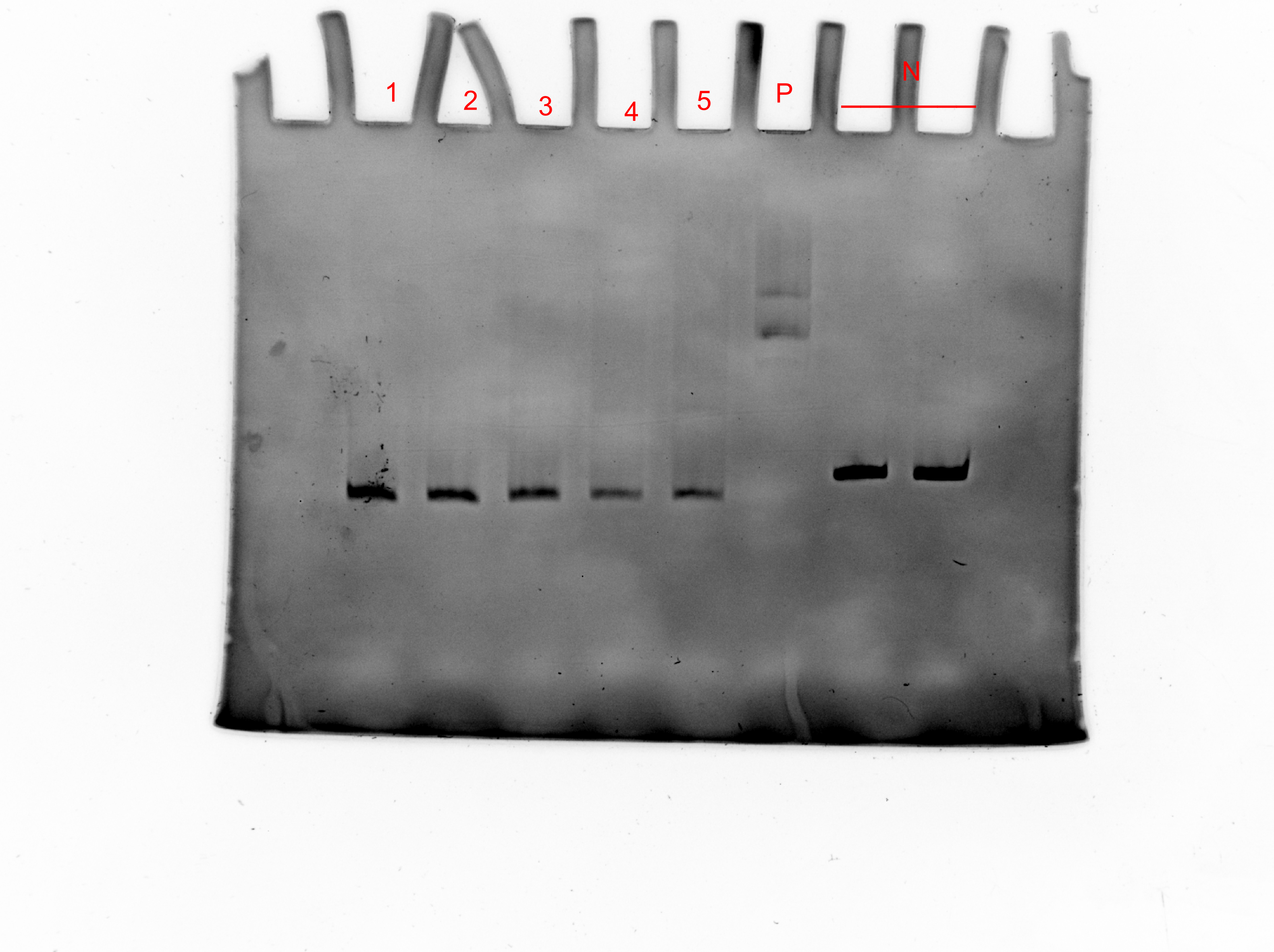

Supplement: S2 Data — Two text files containing all the amino acid sequences for Fig 6E and 6F. (ZIP) [file ppat.1012169.s002.zip › S2_Data/Figure 3E.tif]

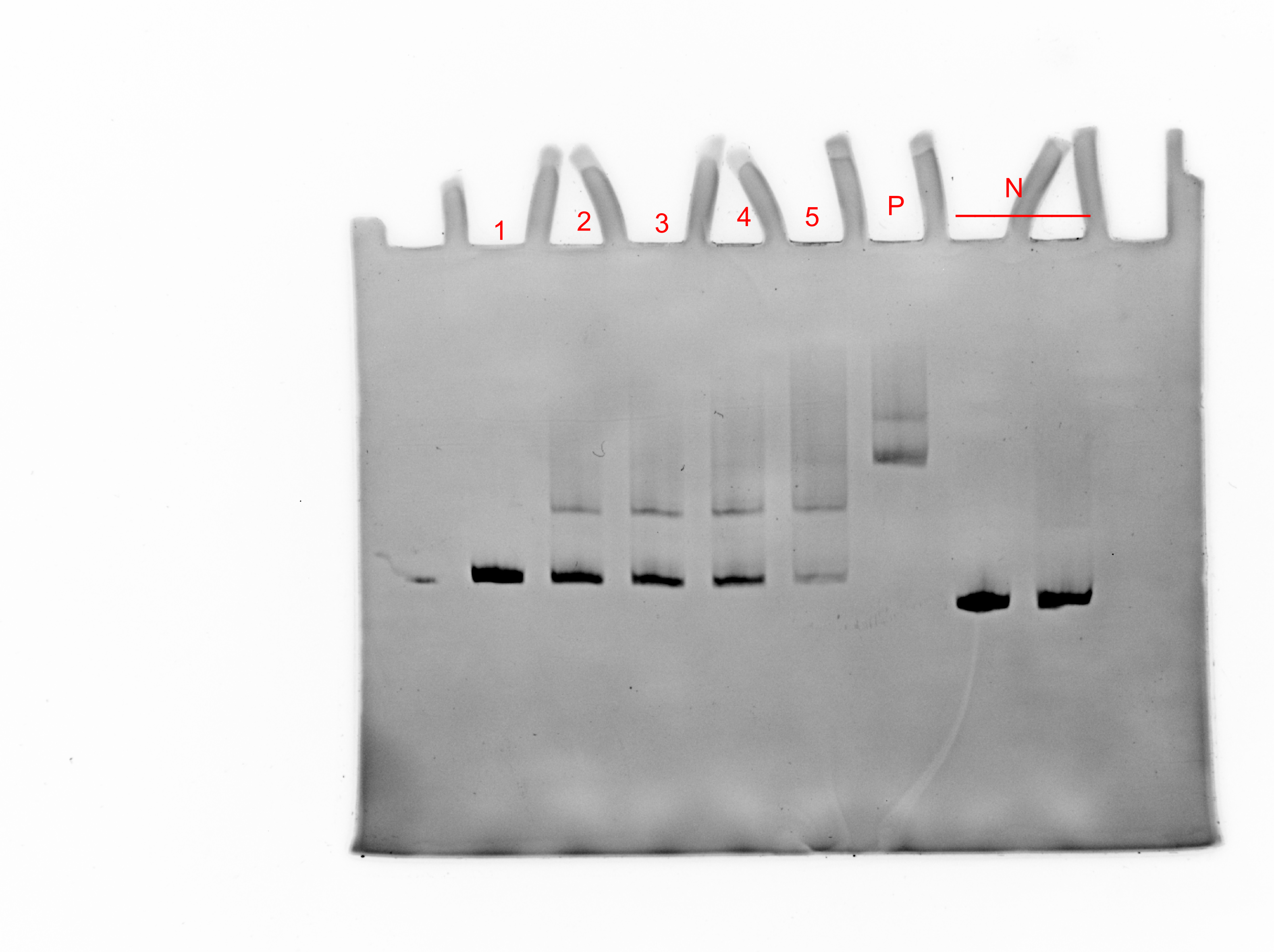

Supplement: S2 Data — Two text files containing all the amino acid sequences for Fig 6E and 6F. (ZIP) [file ppat.1012169.s002.zip › S2_Data/Figure 3H.tif]

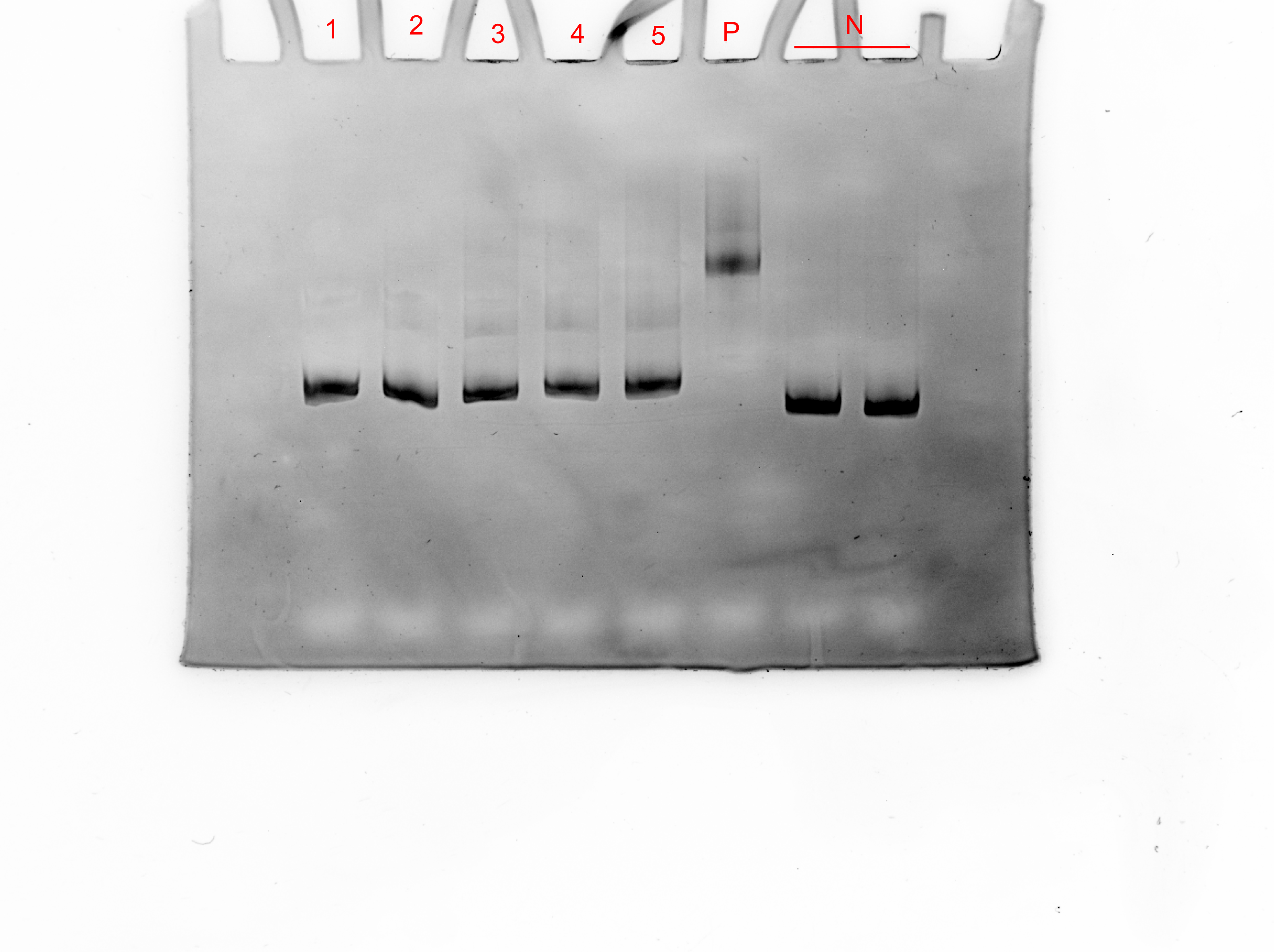

Supplement: S2 Data — Two text files containing all the amino acid sequences for Fig 6E and 6F. (ZIP) [file ppat.1012169.s002.zip › S2_Data/Figure 3I.tif]

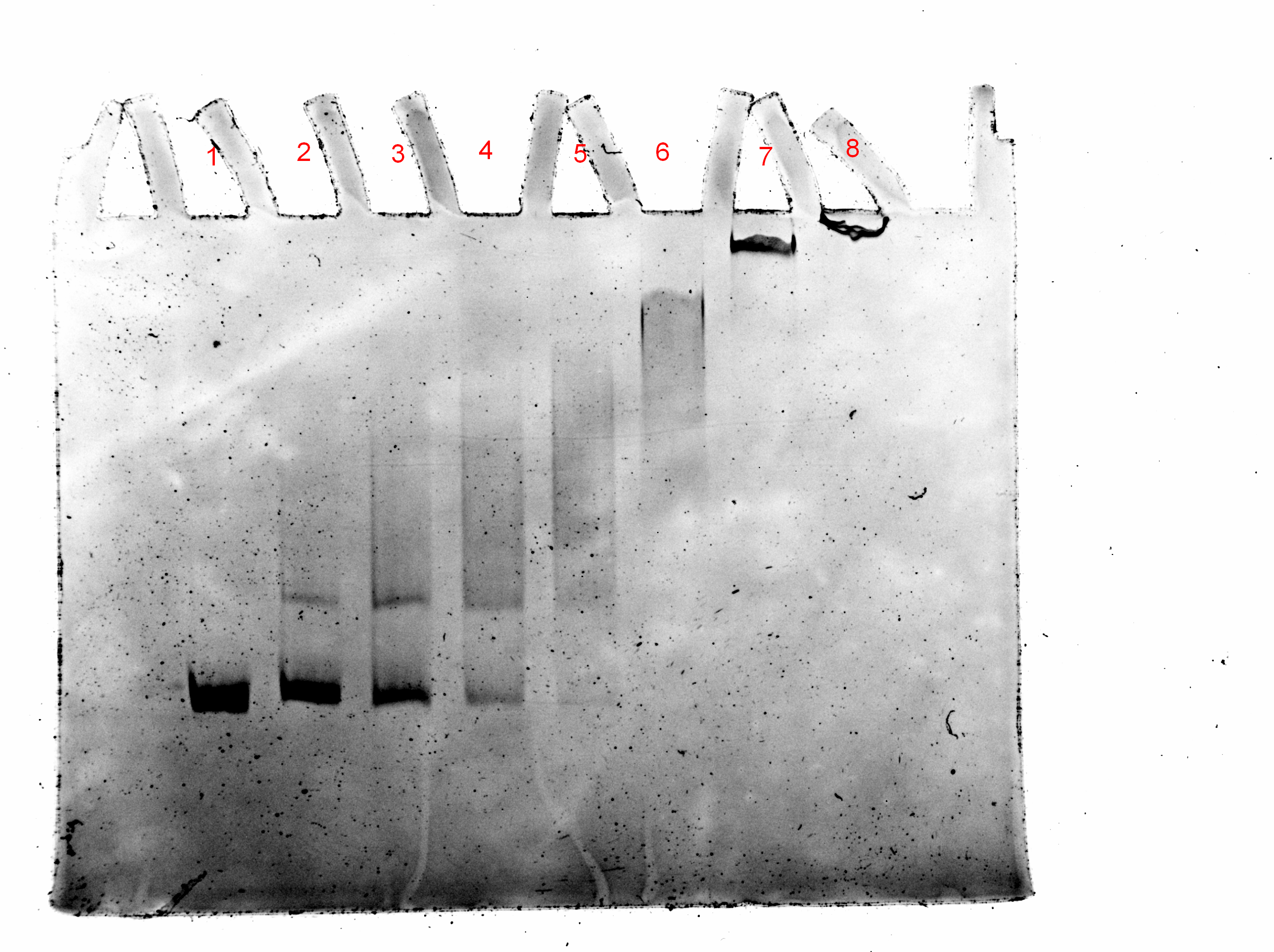

Supplement: S2 Data — Two text files containing all the amino acid sequences for Fig 6E and 6F. (ZIP) [file ppat.1012169.s002.zip › S2_Data/Figure 3J.tif]

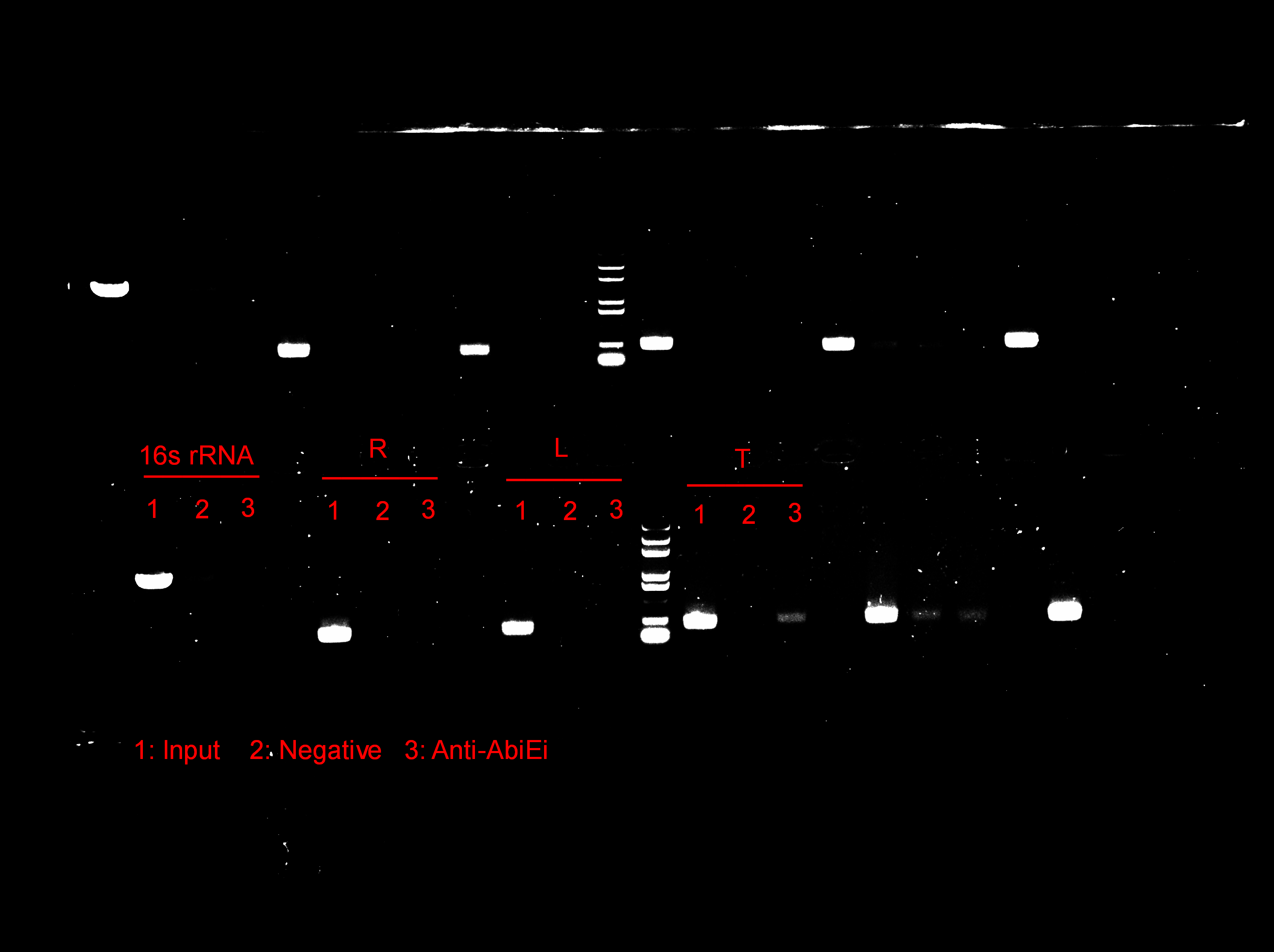

Supplement: S2 Data — Two text files containing all the amino acid sequences for Fig 6E and 6F. (ZIP) [file ppat.1012169.s002.zip › S2_Data/Figure 3K.tif]

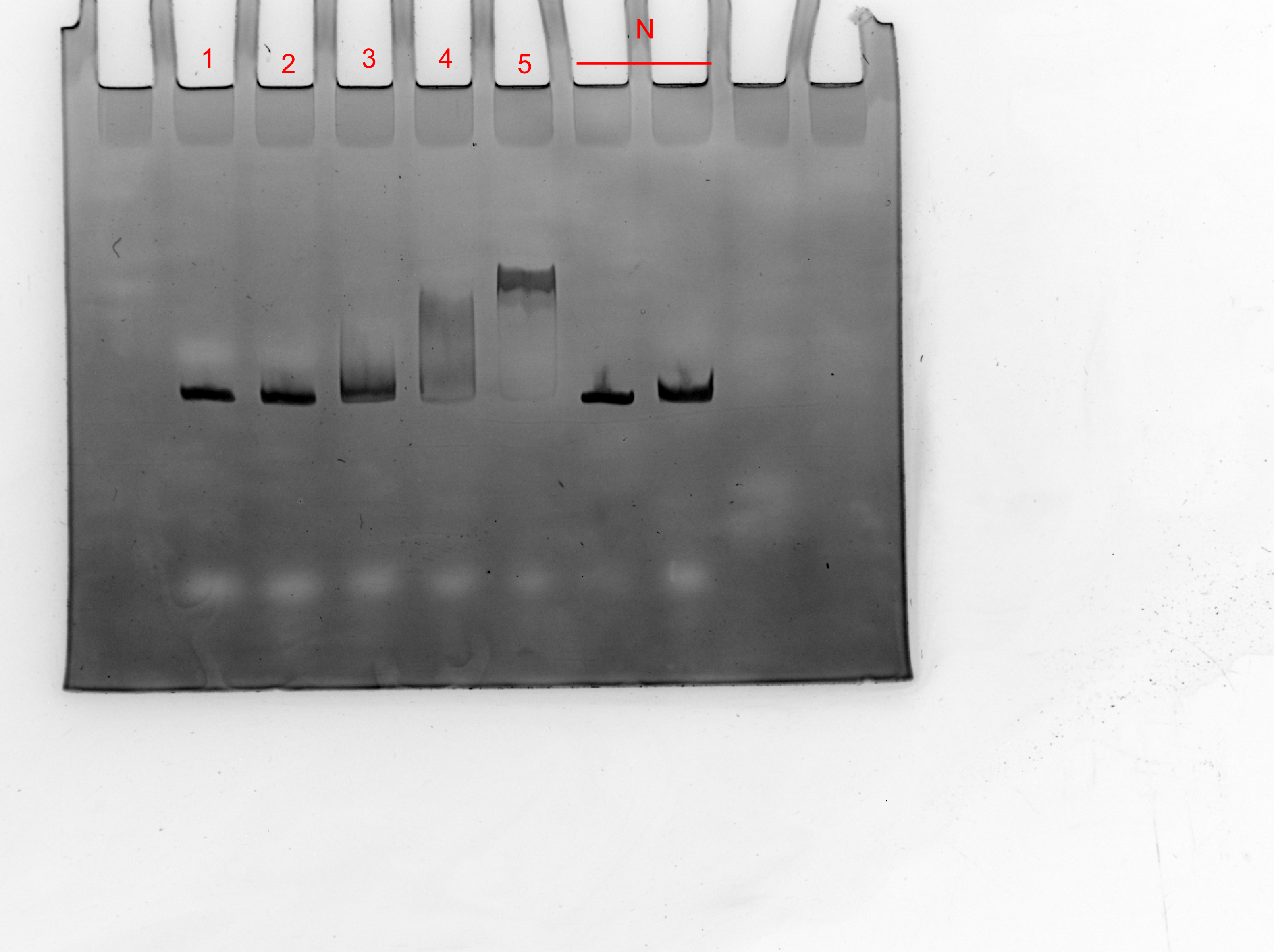

Supplement: S2 Data — Two text files containing all the amino acid sequences for Fig 6E and 6F. (ZIP) [file ppat.1012169.s002.zip › S2_Data/Figure 4B.tif]

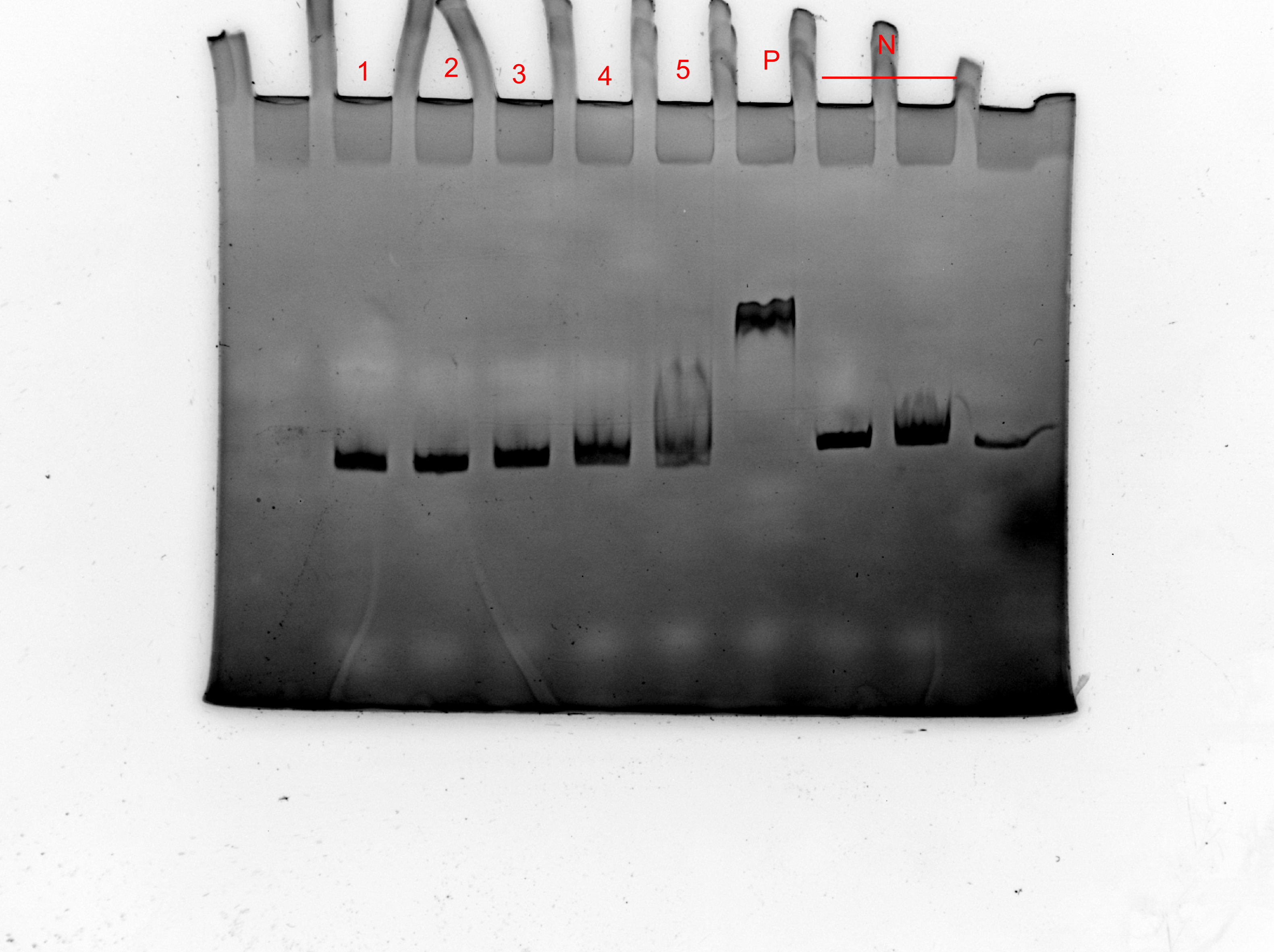

Supplement: S2 Data — Two text files containing all the amino acid sequences for Fig 6E and 6F. (ZIP) [file ppat.1012169.s002.zip › S2_Data/Figure 4C.tif]

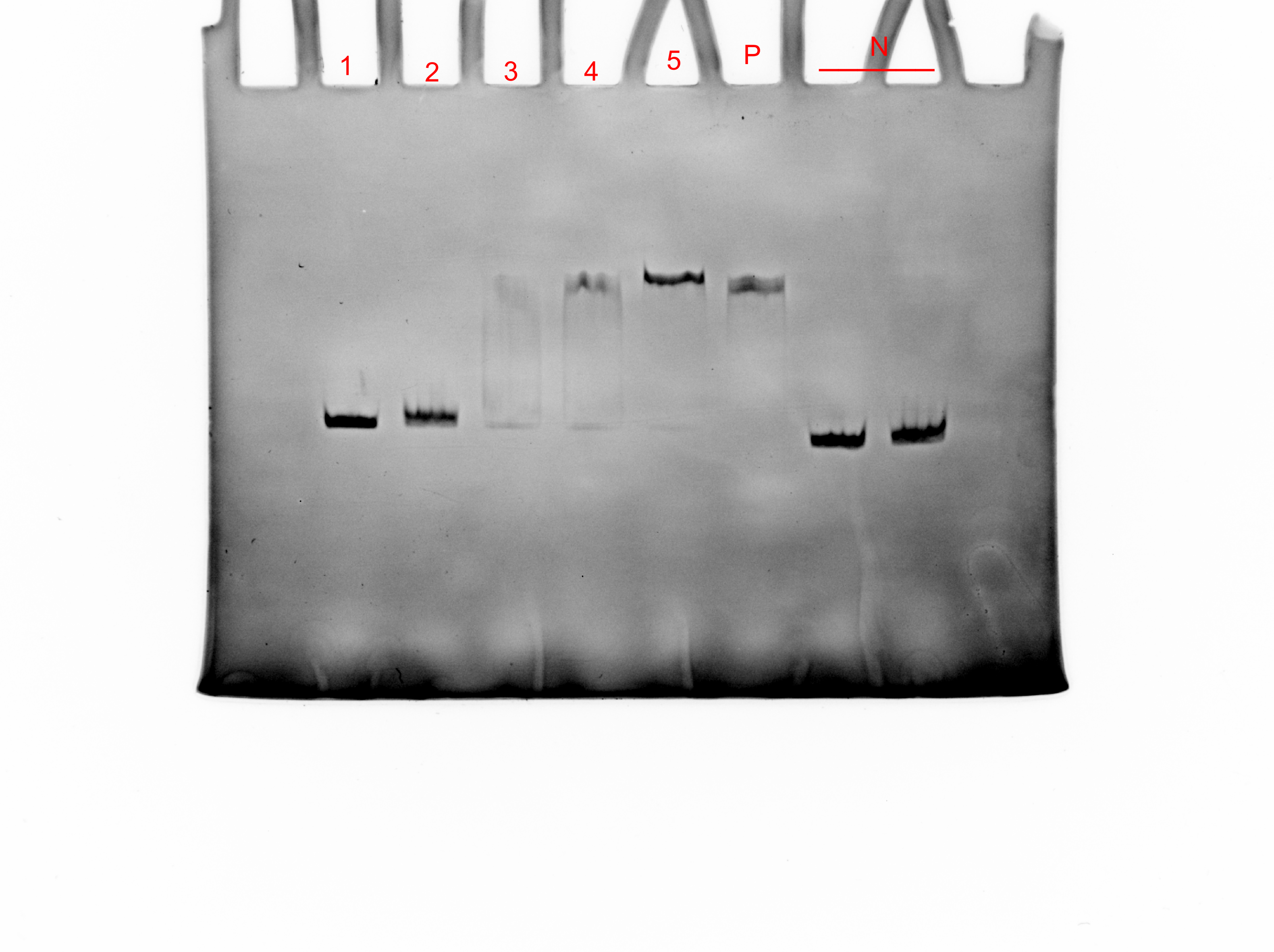

Supplement: S2 Data — Two text files containing all the amino acid sequences for Fig 6E and 6F. (ZIP) [file ppat.1012169.s002.zip › S2_Data/Figure 4F.tif]

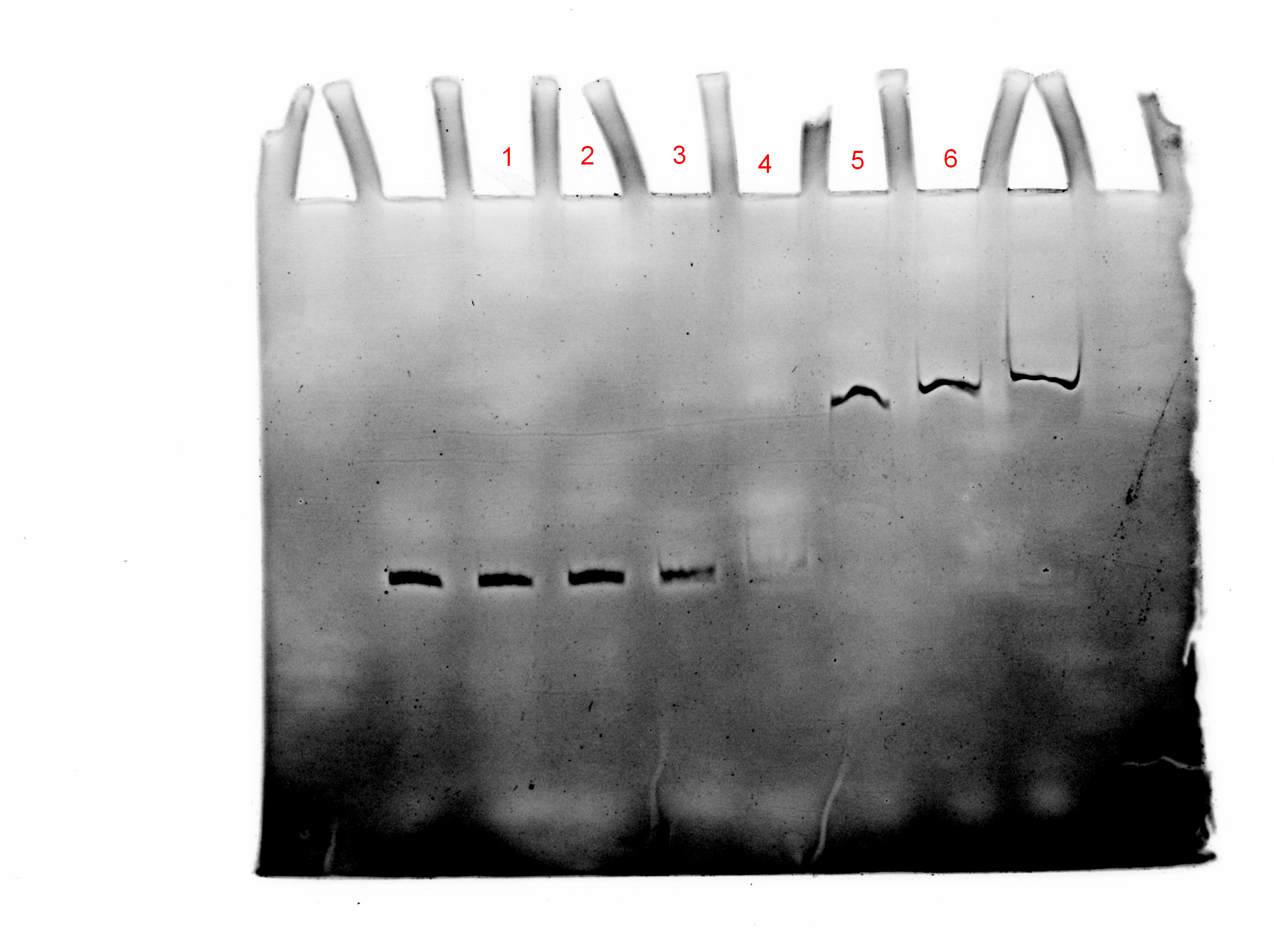

Supplement: S2 Data — Two text files containing all the amino acid sequences for Fig 6E and 6F. (ZIP) [file ppat.1012169.s002.zip › S2_Data/Figure 4G.tif]

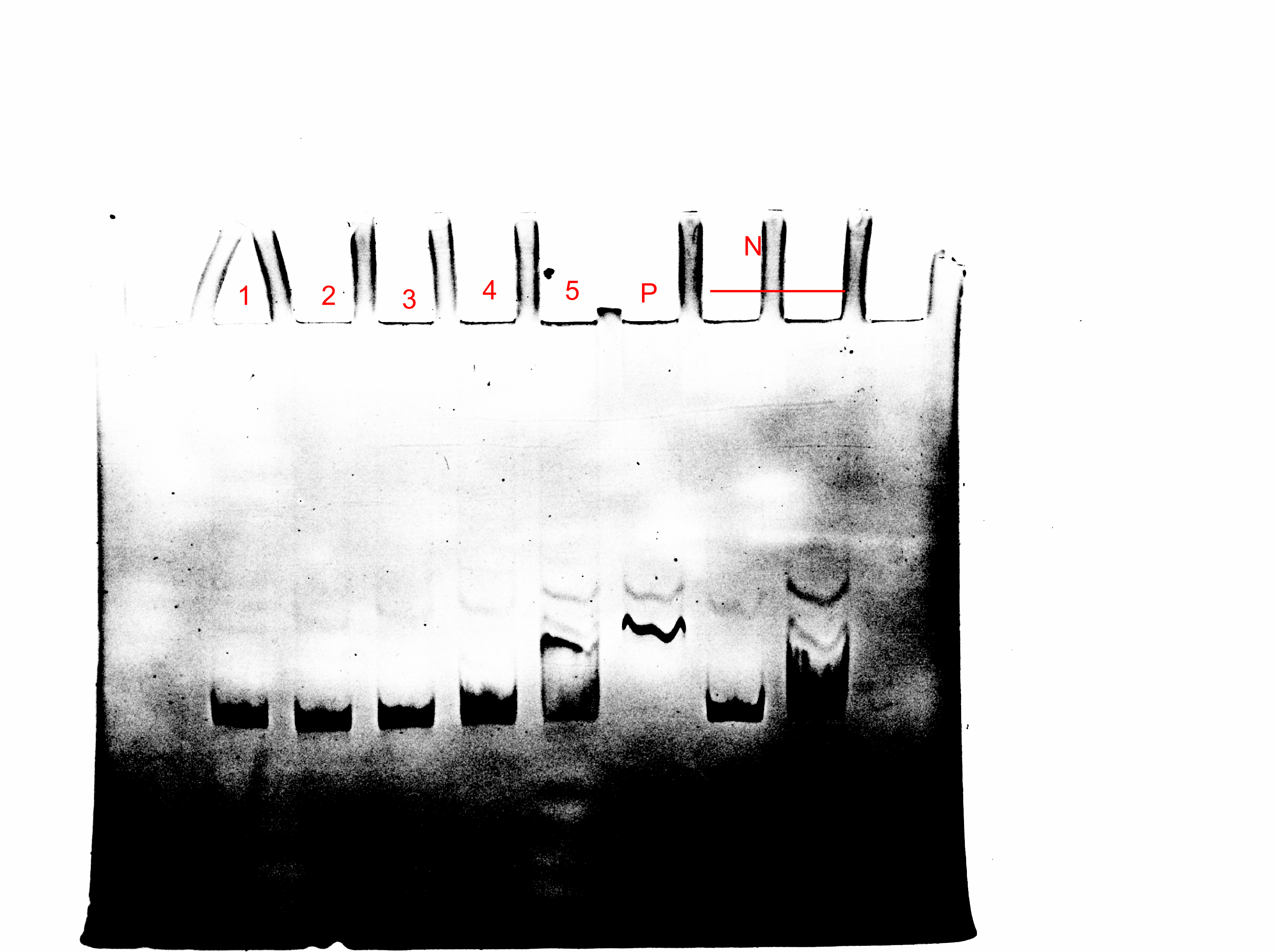

Supplement: S2 Data — Two text files containing all the amino acid sequences for Fig 6E and 6F. (ZIP) [file ppat.1012169.s002.zip › S2_Data/Figure 4H.tif]

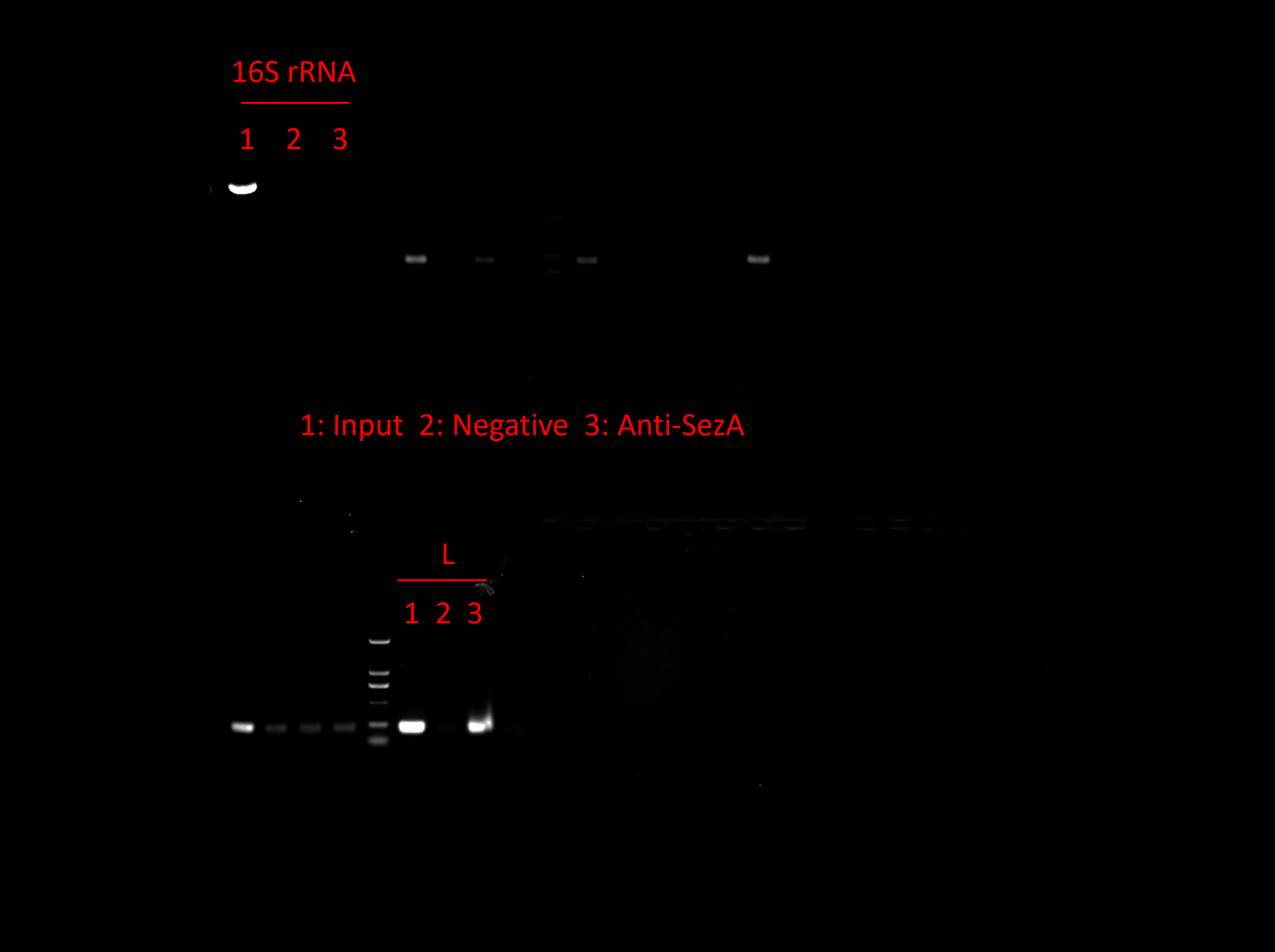

Supplement: S2 Data — Two text files containing all the amino acid sequences for Fig 6E and 6F. (ZIP) [file ppat.1012169.s002.zip › S2_Data/Figure 4I (L).tif]

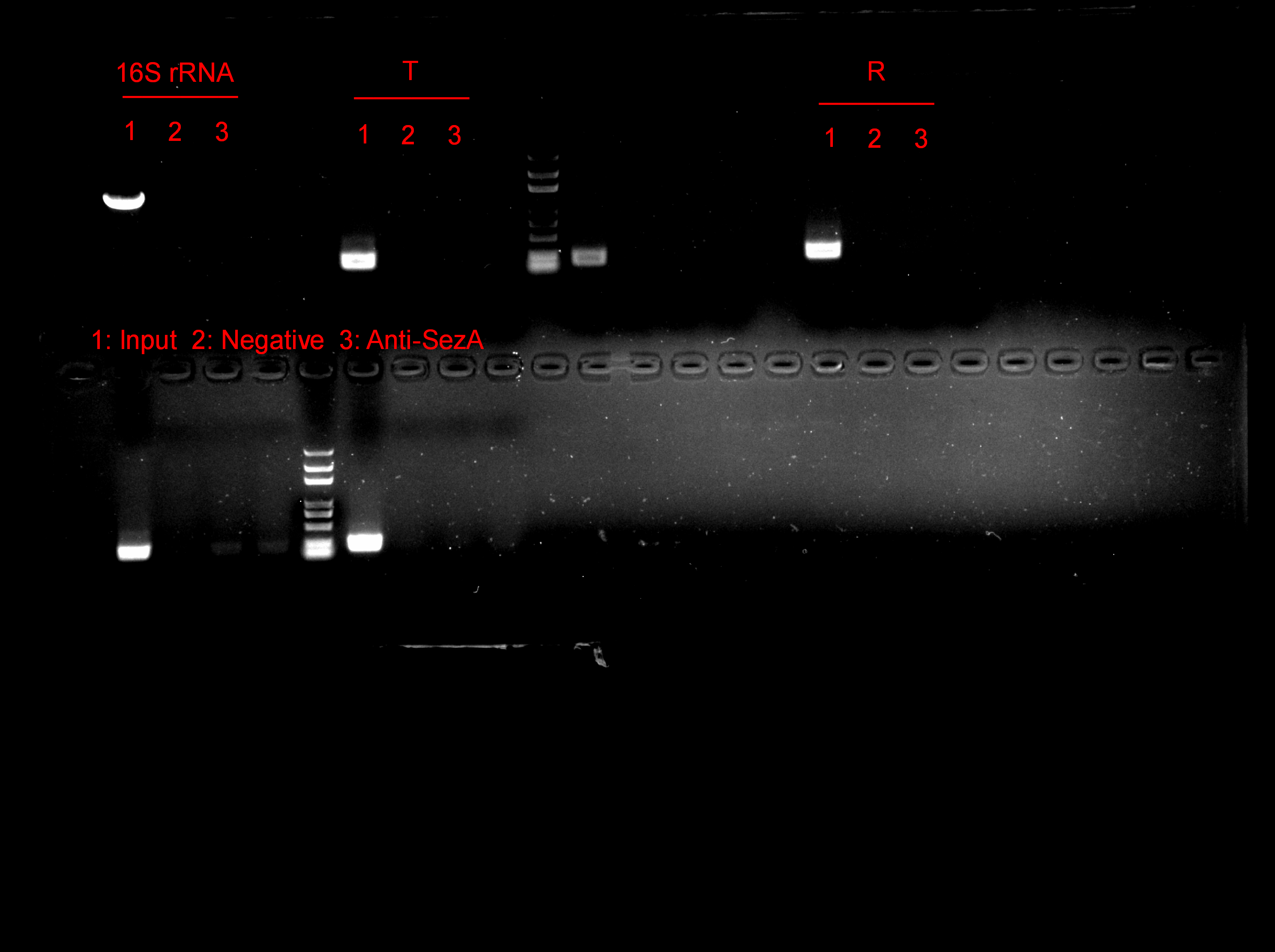

Supplement: S2 Data — Two text files containing all the amino acid sequences for Fig 6E and 6F. (ZIP) [file ppat.1012169.s002.zip › S2_Data/Figure 4I(16S rRNAíóTíóR).tif]

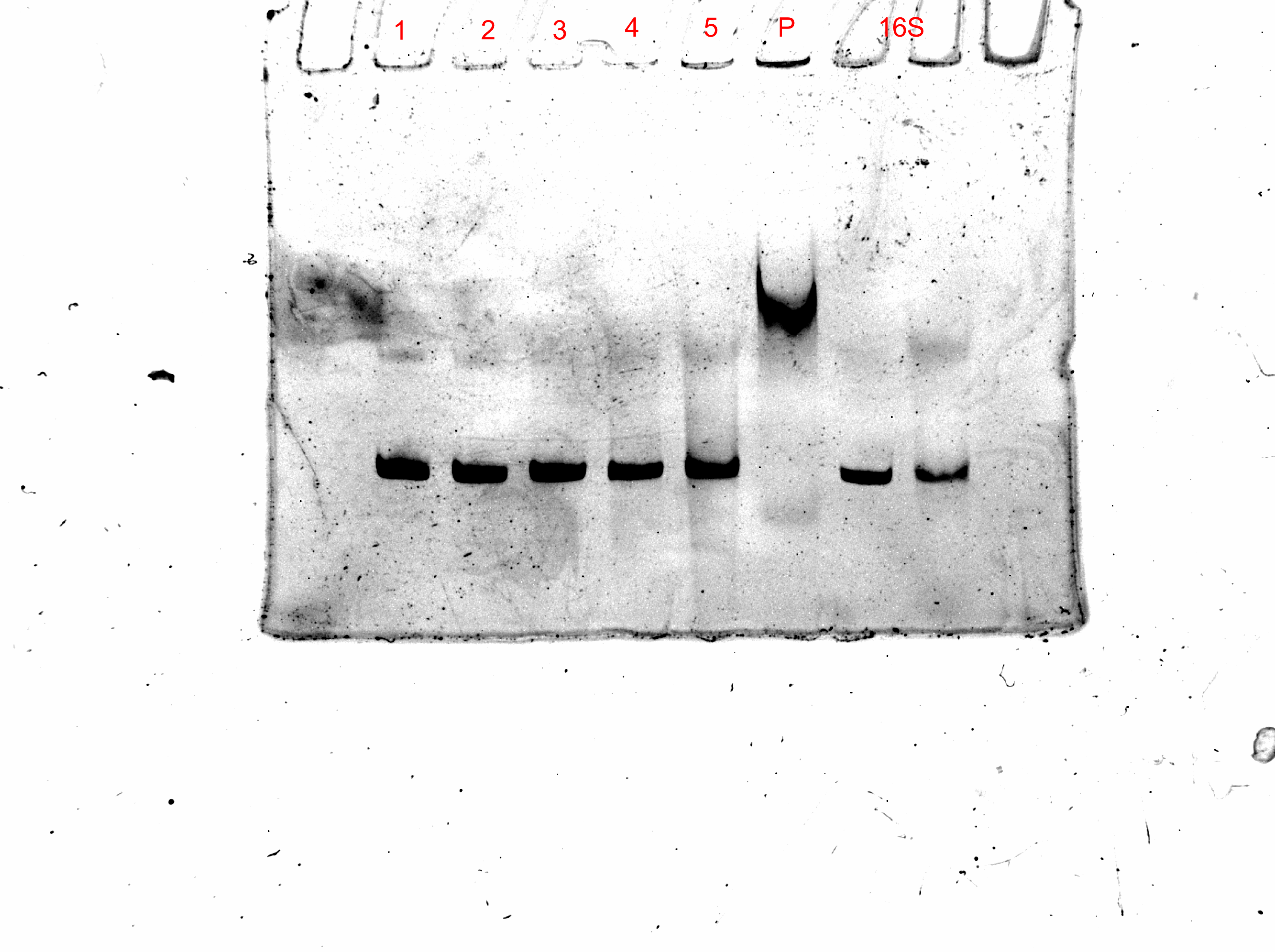

Supplement: S2 Data — Two text files containing all the amino acid sequences for Fig 6E and 6F. (ZIP) [file ppat.1012169.s002.zip › S2_Data/Figure 5E.tif]

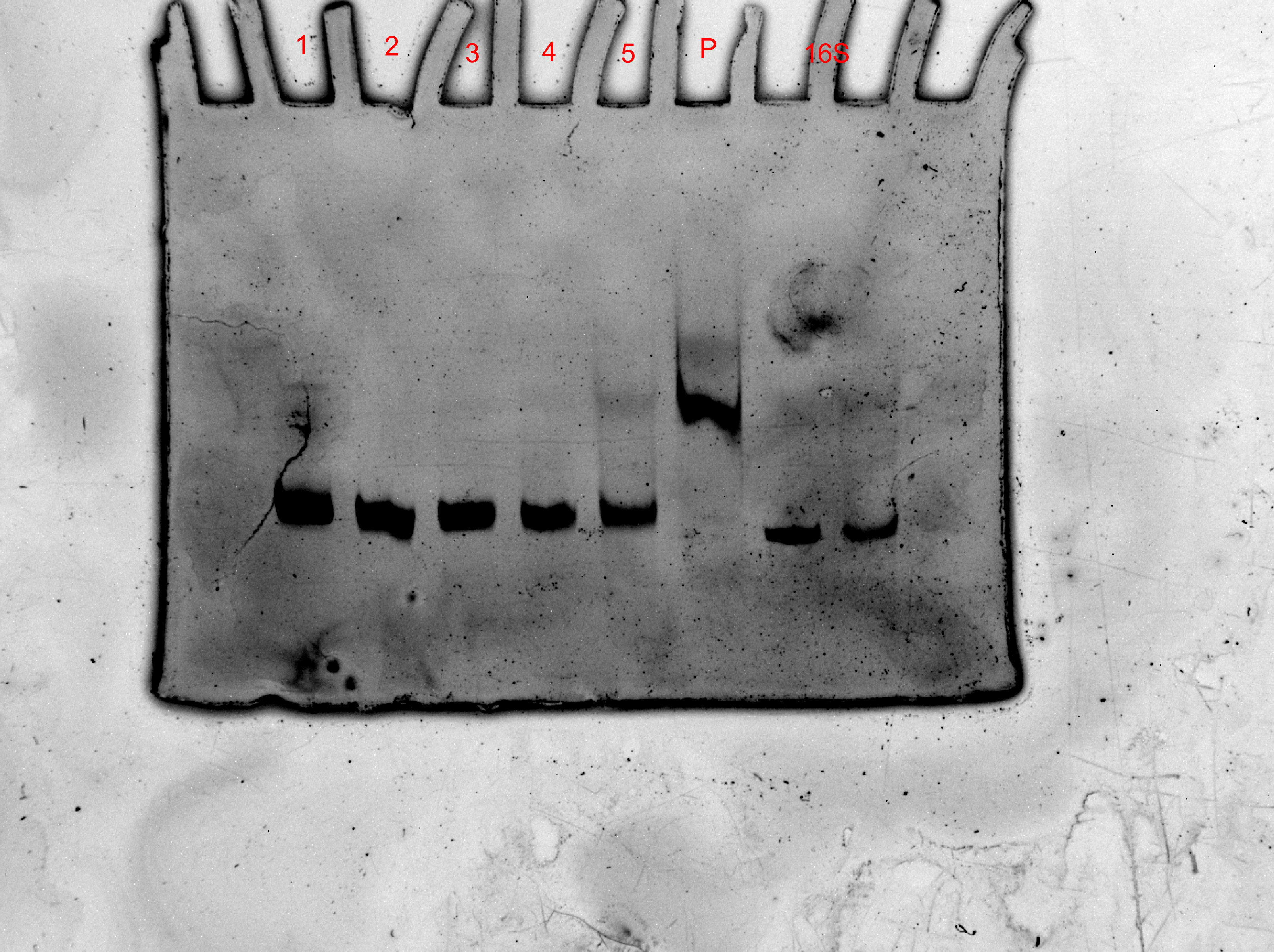

Supplement: S2 Data — Two text files containing all the amino acid sequences for Fig 6E and 6F. (ZIP) [file ppat.1012169.s002.zip › S2_Data/Figure 5F.tif]

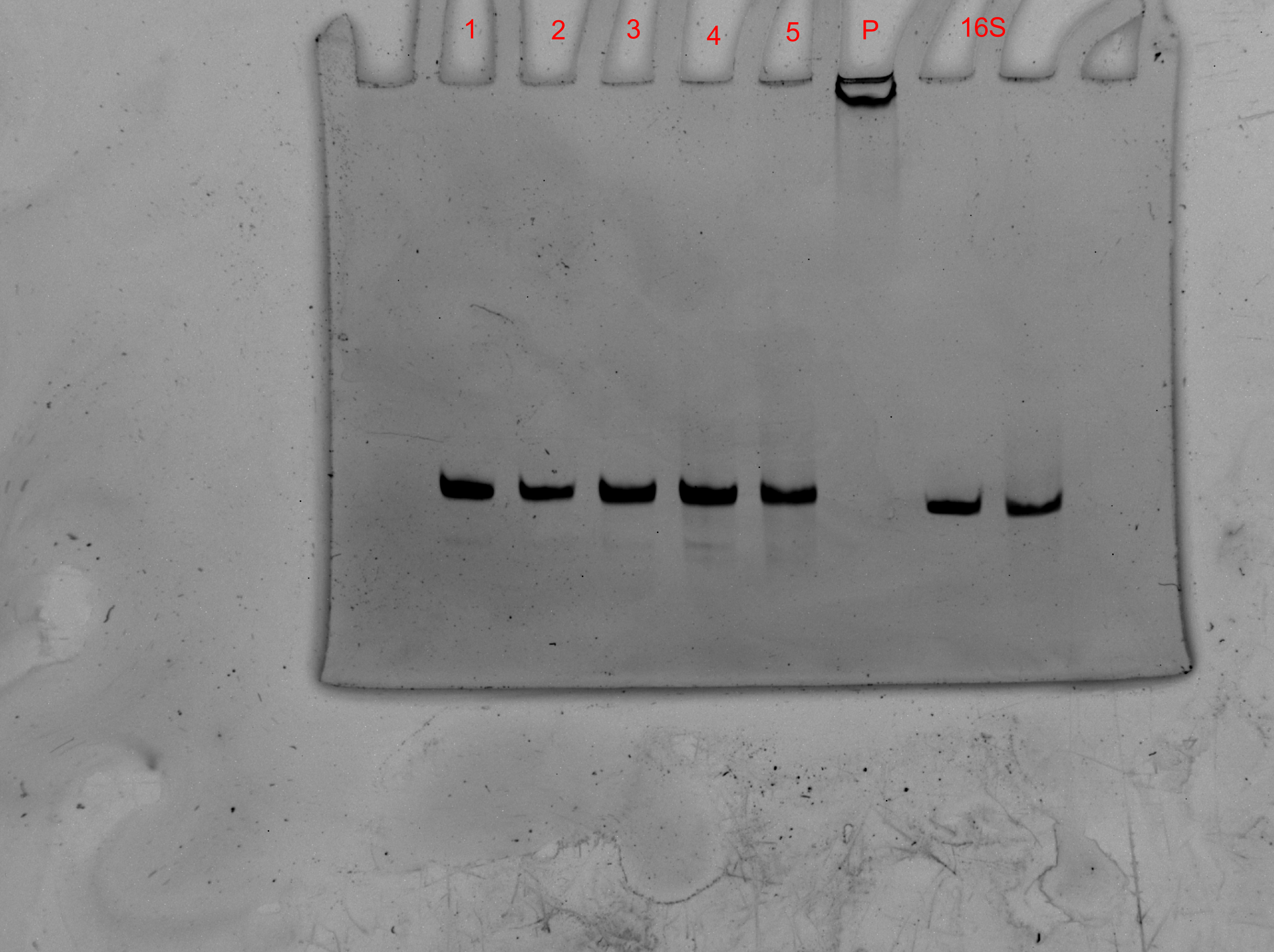

Supplement: S2 Data — Two text files containing all the amino acid sequences for Fig 6E and 6F. (ZIP) [file ppat.1012169.s002.zip › S2_Data/Figure 5G.tif]

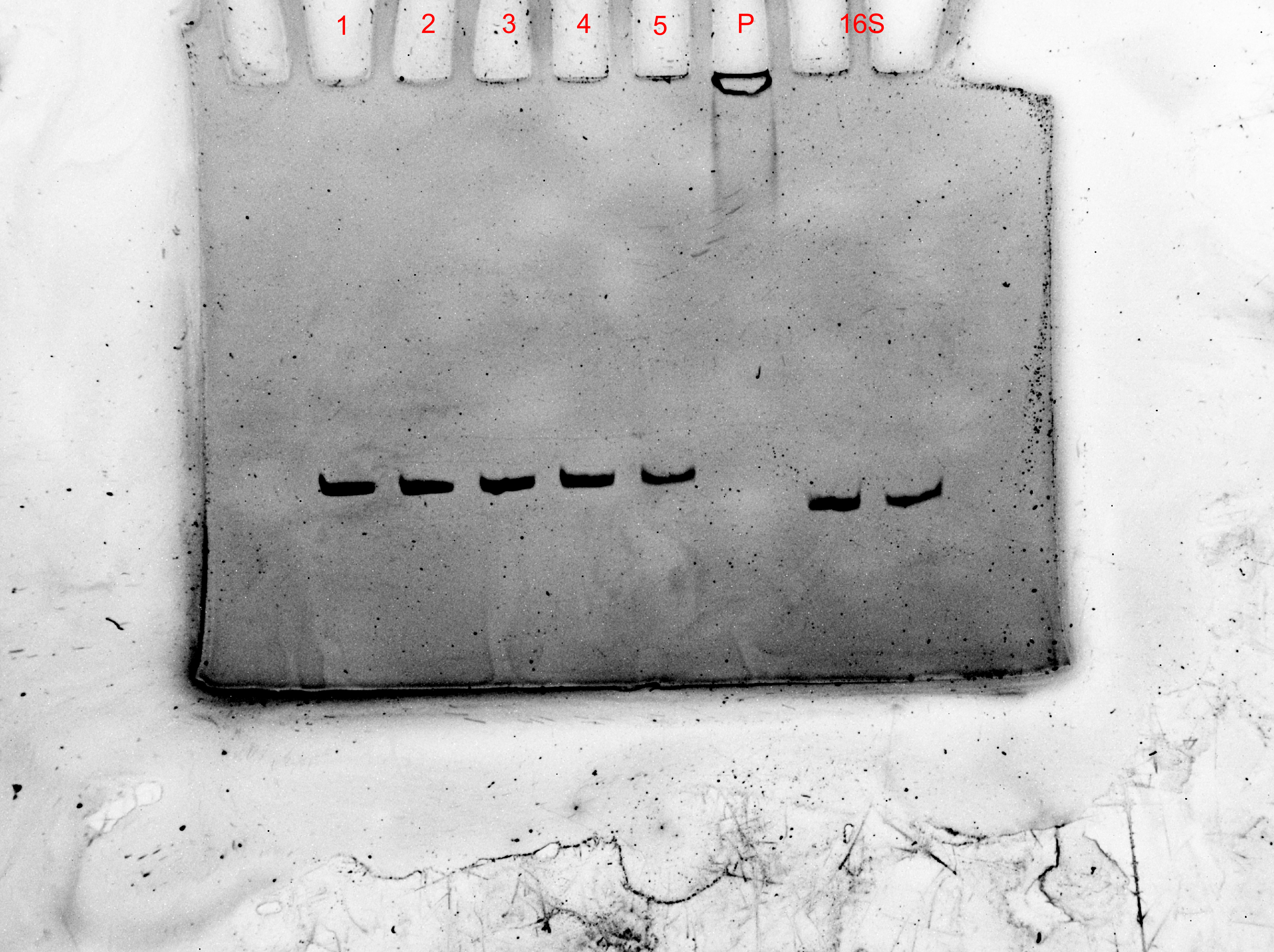

Supplement: S2 Data — Two text files containing all the amino acid sequences for Fig 6E and 6F. (ZIP) [file ppat.1012169.s002.zip › S2_Data/Figure 5H.tif]

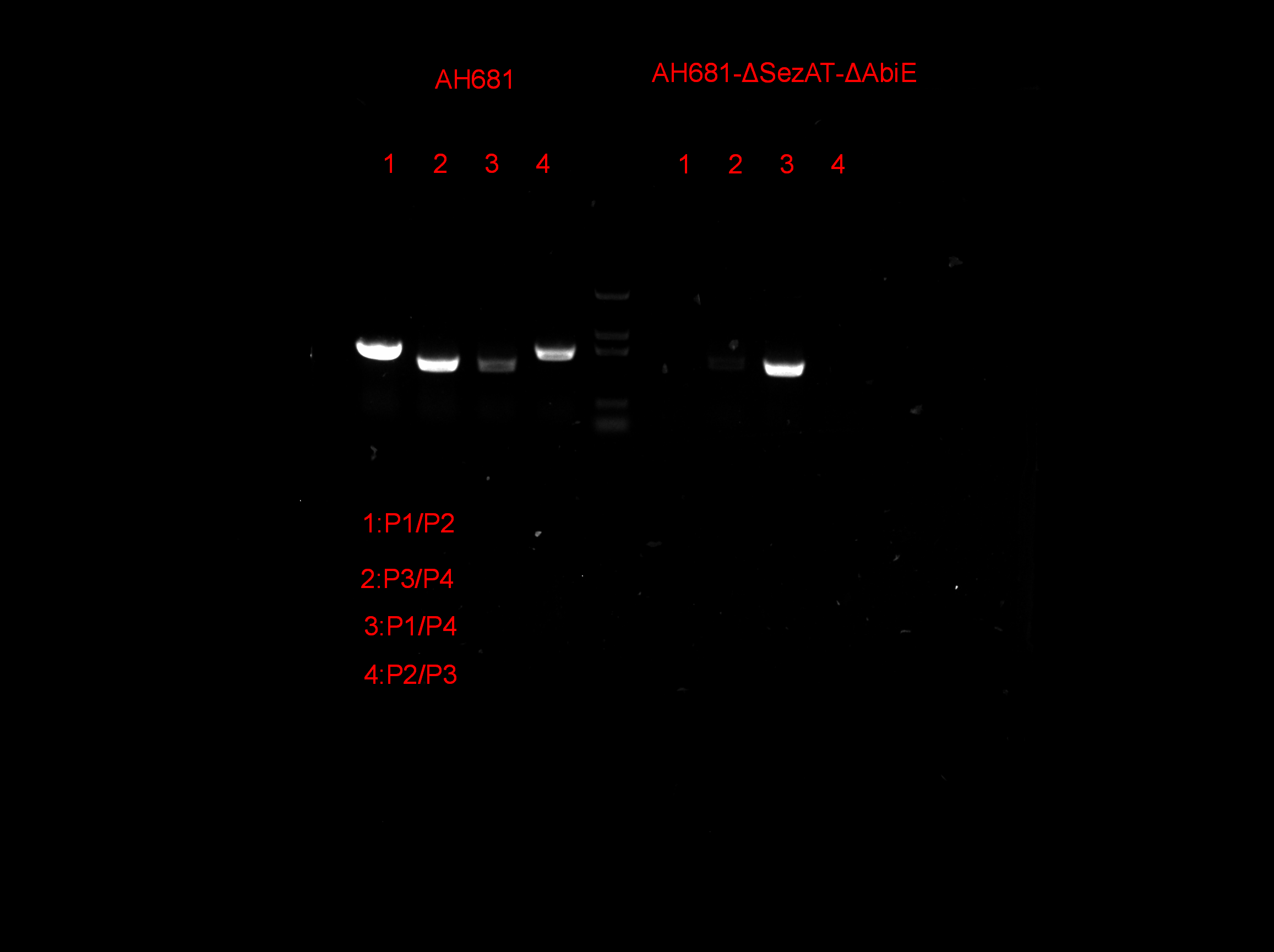

Supplement: S2 Data — Two text files containing all the amino acid sequences for Fig 6E and 6F. (ZIP) [file ppat.1012169.s002.zip › S2_Data/Figure 6I.tif]

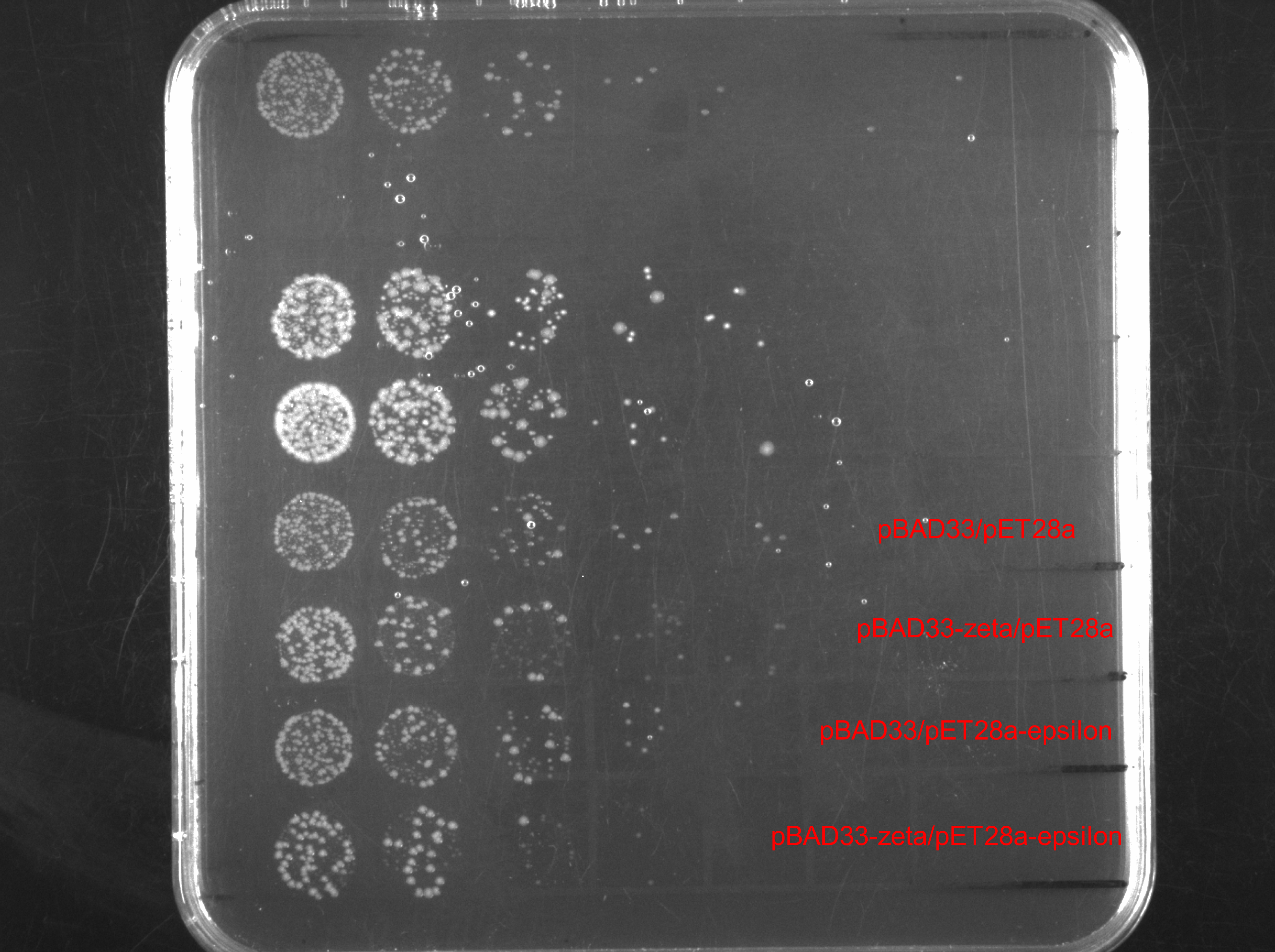

Supplement: S2 Data — Two text files containing all the amino acid sequences for Fig 6E and 6F. (ZIP) [file ppat.1012169.s002.zip › S2_Data/Figure S1A.tif]

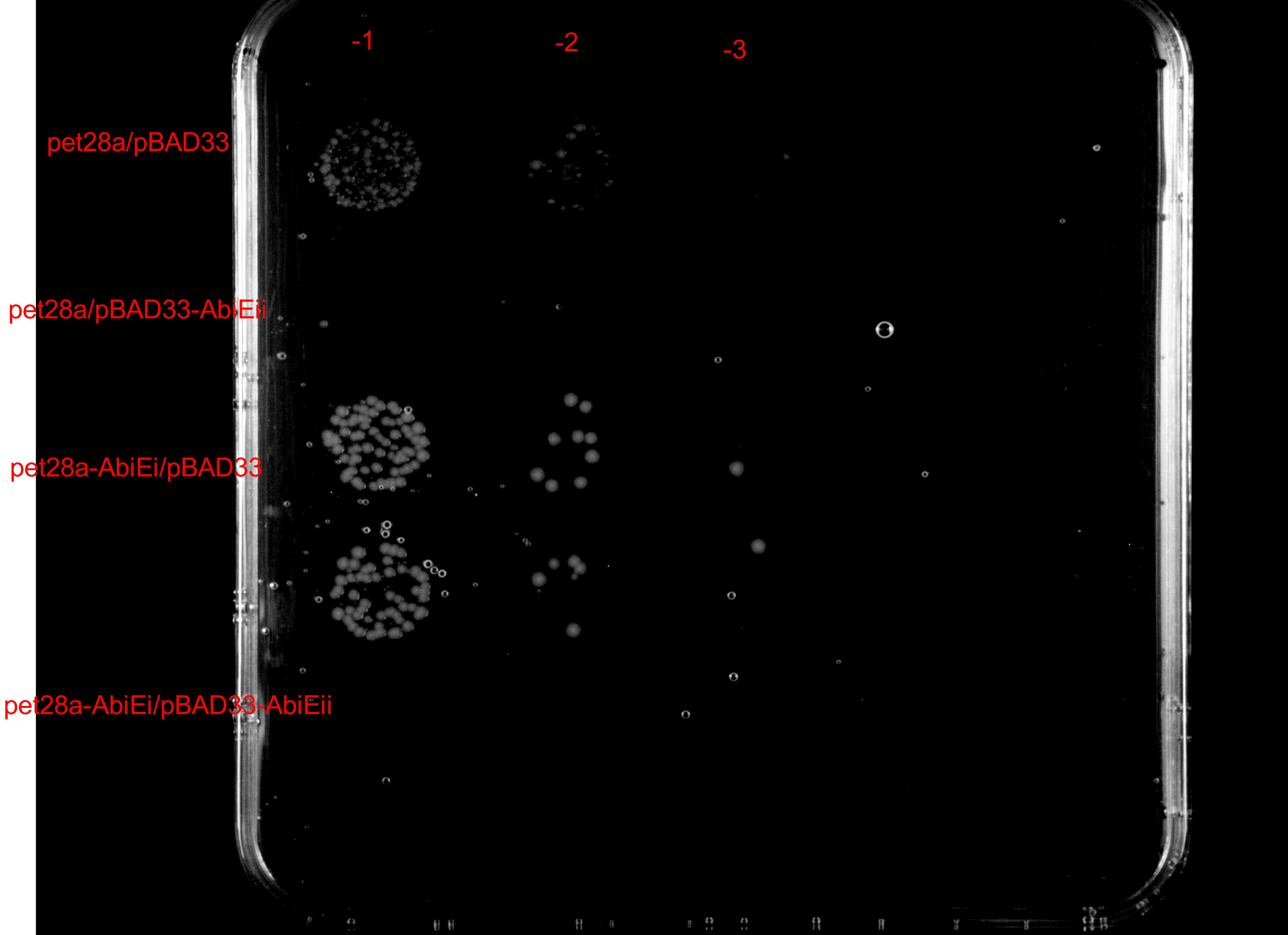

Supplement: S2 Data — Two text files containing all the amino acid sequences for Fig 6E and 6F. (ZIP) [file ppat.1012169.s002.zip › S2_Data/Figure S1E.tif]

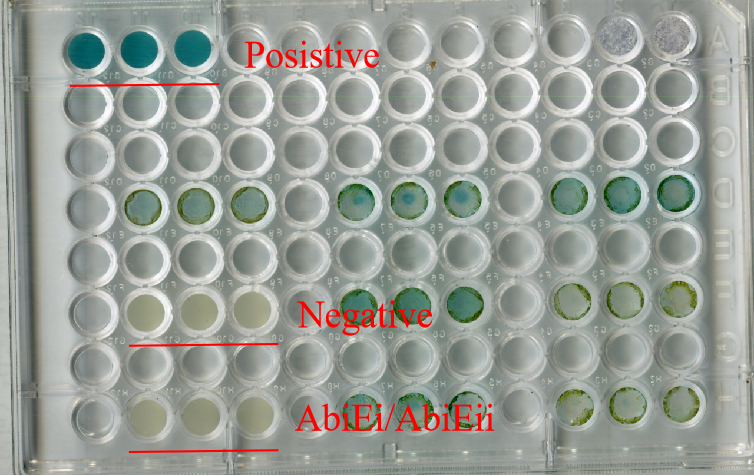

Supplement: S2 Data — Two text files containing all the amino acid sequences for Fig 6E and 6F. (ZIP) [file ppat.1012169.s002.zip › S2_Data/Figure S1G.tif]

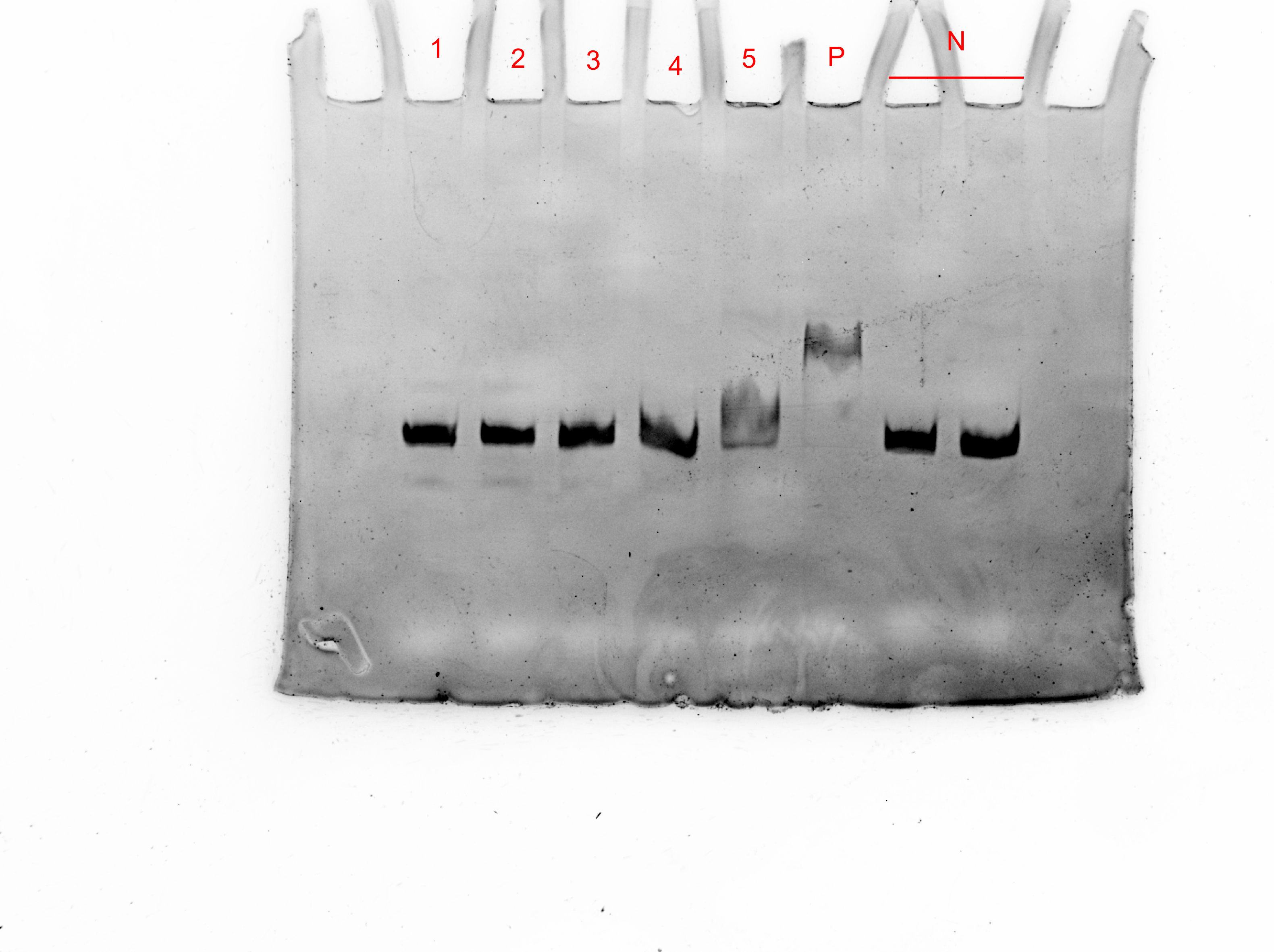

Supplement: S2 Data — Two text files containing all the amino acid sequences for Fig 6E and 6F. (ZIP) [file ppat.1012169.s002.zip › S2_Data/Figure S7C.tif]

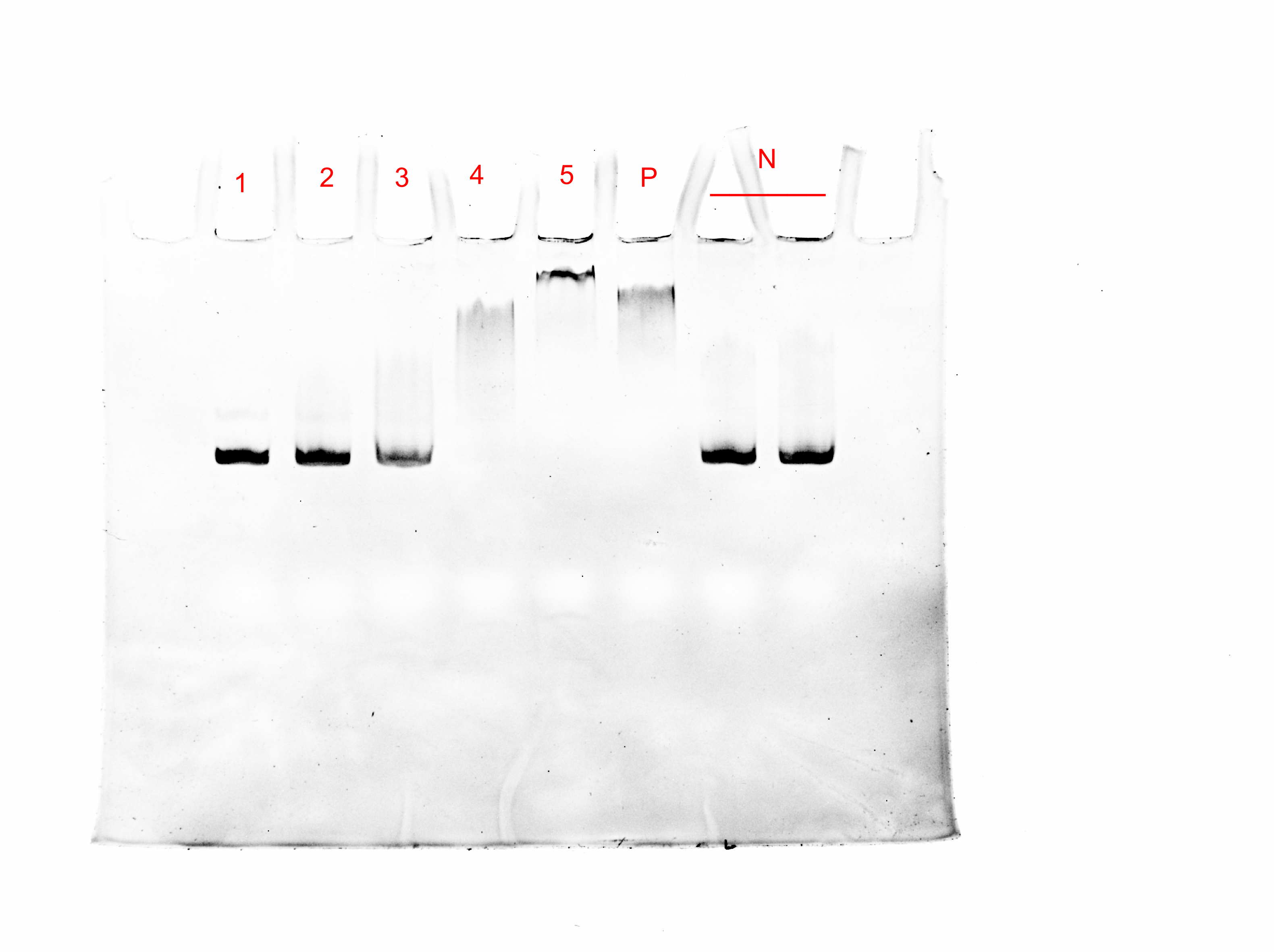

Supplement: S2 Data — Two text files containing all the amino acid sequences for Fig 6E and 6F. (ZIP) [file ppat.1012169.s002.zip › S2_Data/Figure S7E.tif]

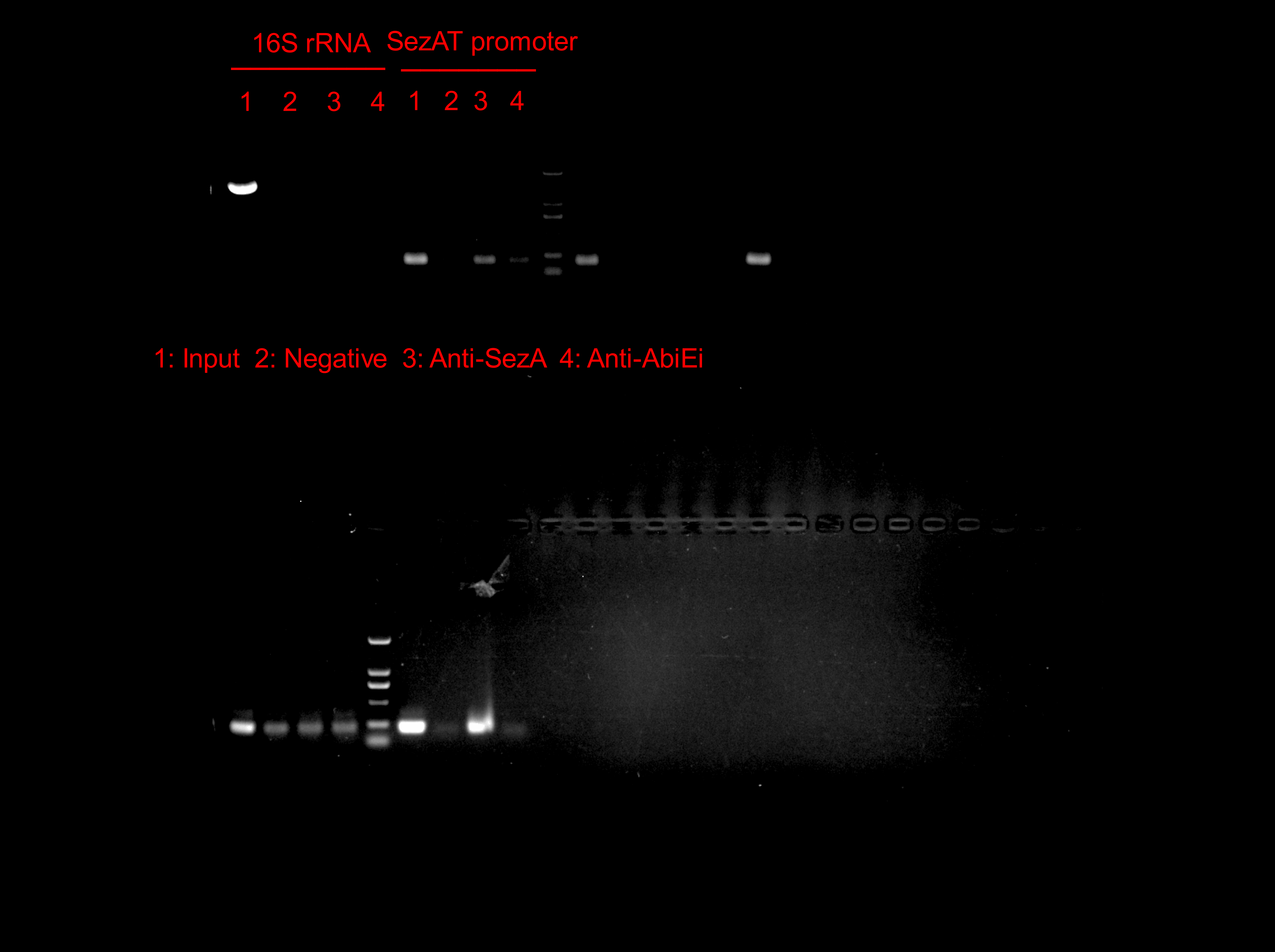

Supplement: S2 Data — Two text files containing all the amino acid sequences for Fig 6E and 6F. (ZIP) [file ppat.1012169.s002.zip › S2_Data/Figure S7F.tif]

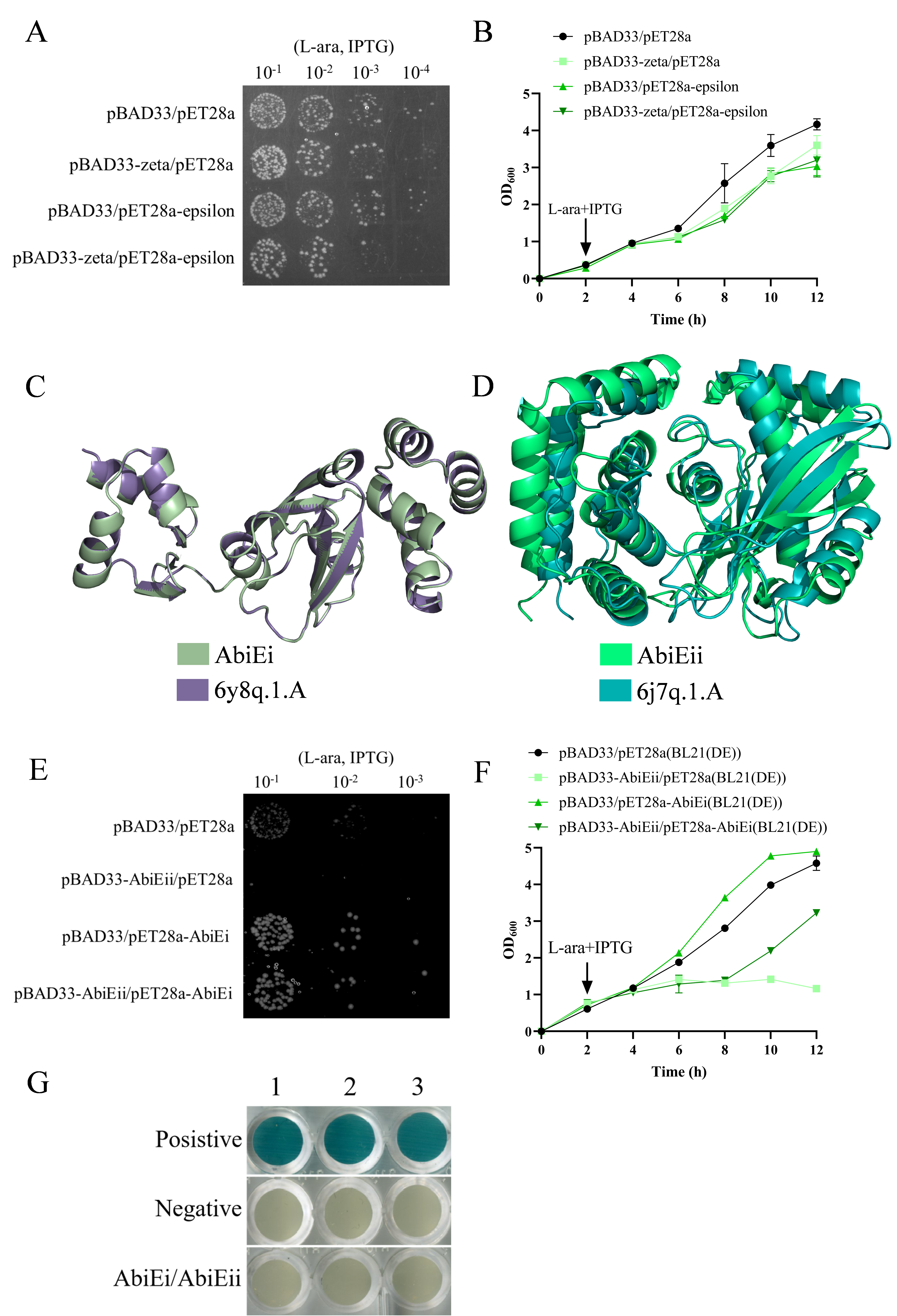

Supplement: S1 Fig — (A) CFUs were analyzed for the toxicity of zeta and the ability of epsilon to neutralize toxicity. (B) Growth curves for cells transfected with pBAD33/pET28a, pBAD33-zeta/pET28a, pBAD33/pET28a-epsilon or pBAD33-zeta/pET28a-epsilon vectors were determined under L-arabinose and IPTG induction. (C) Tertiary structure alignment of the AbiEi antitoxin in HN105 with the AbiEi antitoxin (6y8q.1.A) in Streptococcus agalactiae. AbiEi in HN105 overlaps almost completely with the tertiary structure of 6y8q.1.A. The amino acid sequence identity between AbiEi in HN105 and 6y8q.1.A is 88.21%. (D) AbiEii in HN105 largely overlaps with the tertiary structure of the TglT toxin (guanylytransferase-like toxin, 6j7q.1.A) in Mycobacterium tuberculosis. The amino acid sequence identity between AbiEii and 6j7q.1.A is 15.73%. (E) CFUs were analyzed for the toxicity of AbiEii and the ability of AbiEi to neutralize toxicity. (F) CFUs assay for the identification of AbiE activity using pBAD33 and pET28a co-transformation. (G) The interaction between AbiEi and AbIEii was verified by bacterial two-hybrid analysis. 1–3 are three independent replicates. (TIF) [file ppat.1012169.s003.tif]

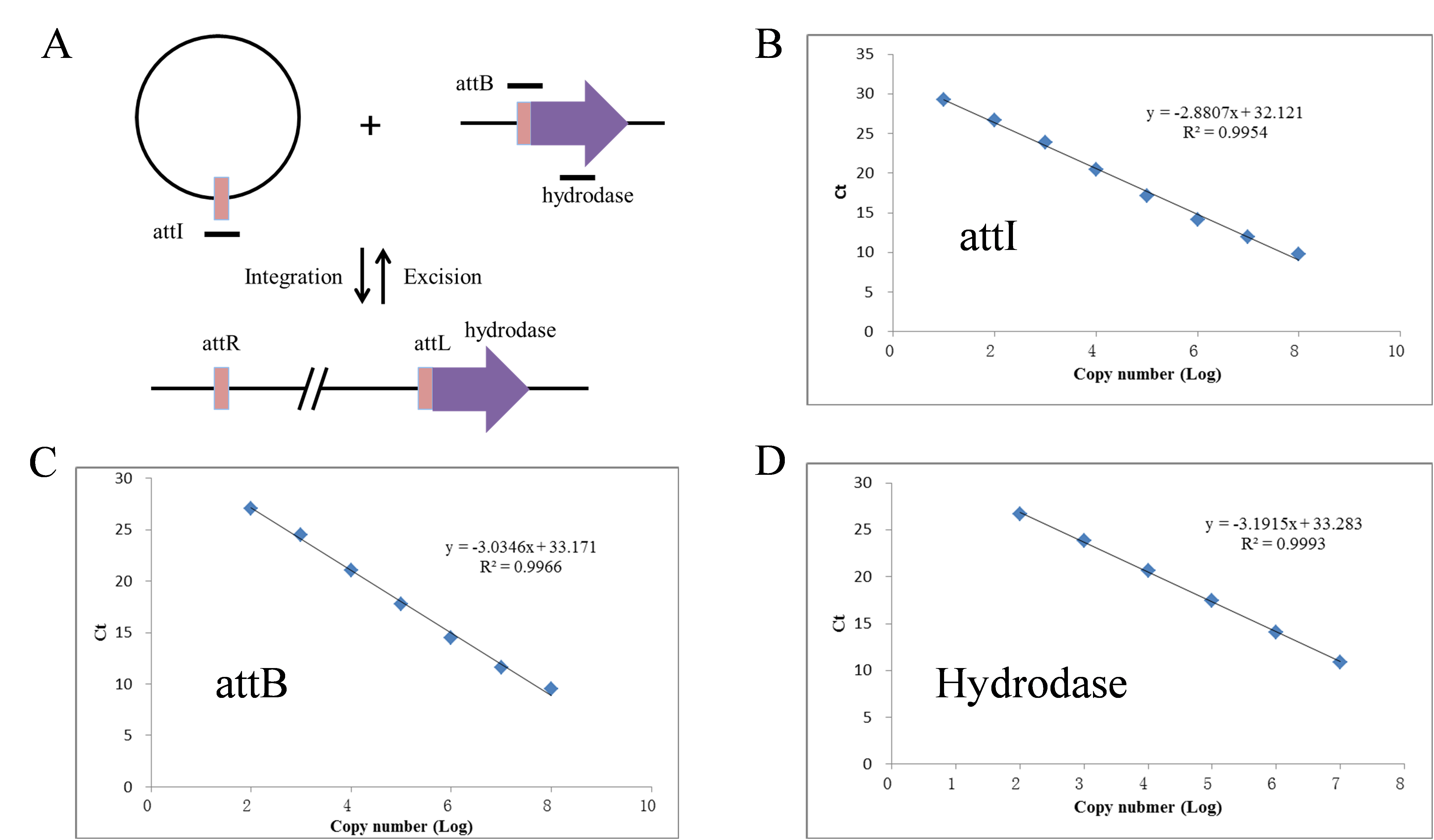

Supplement: S2 Fig — (A) Schematic representation of the excision and integration of ICESsuHN05. Bold black short lines represent the locations of attI, attB and Hydrodase fragment amplification. Standard curves for attI (B), attB (C) and Hydrodase (D) copy number analysis. (TIF) [file ppat.1012169.s004.tif]

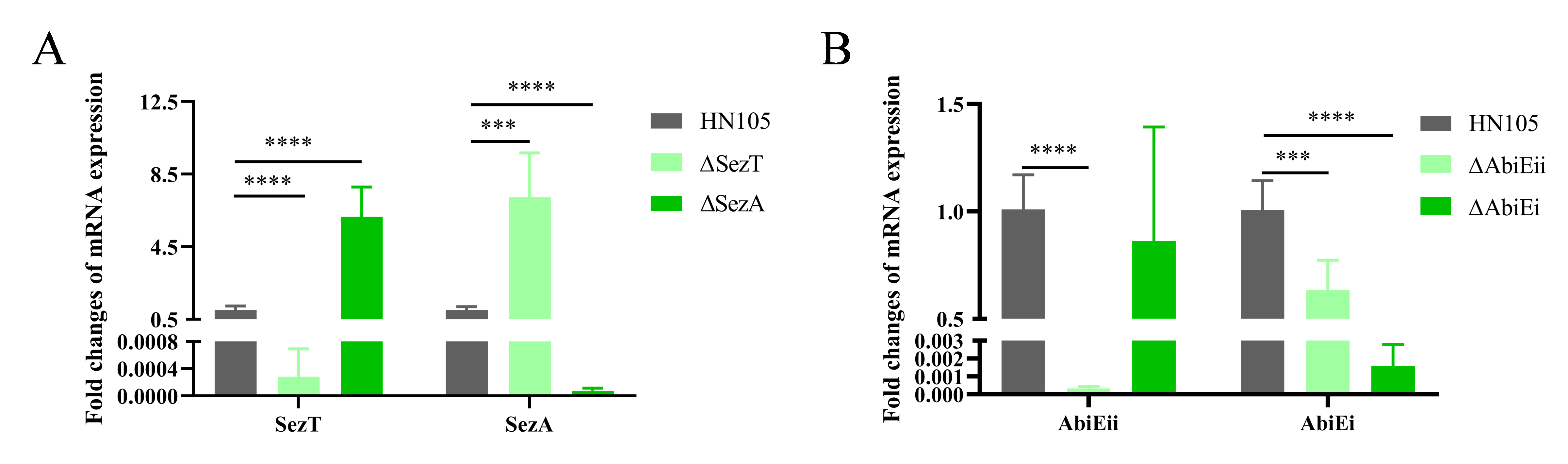

Supplement: S3 Fig — (A) Transcript levels of SezA and SezT were determined in ΔSezA and ΔSezT. (B) Transcript levels of AbiEi and AbiEii were determined in ΔAbiEi and ΔAbiEii. All experiments were conducted independently three times. Unpaired two-tailed Student’s t-test: *** P < 0.001; **** P < 0.0001. (TIF) [file ppat.1012169.s005.tif]

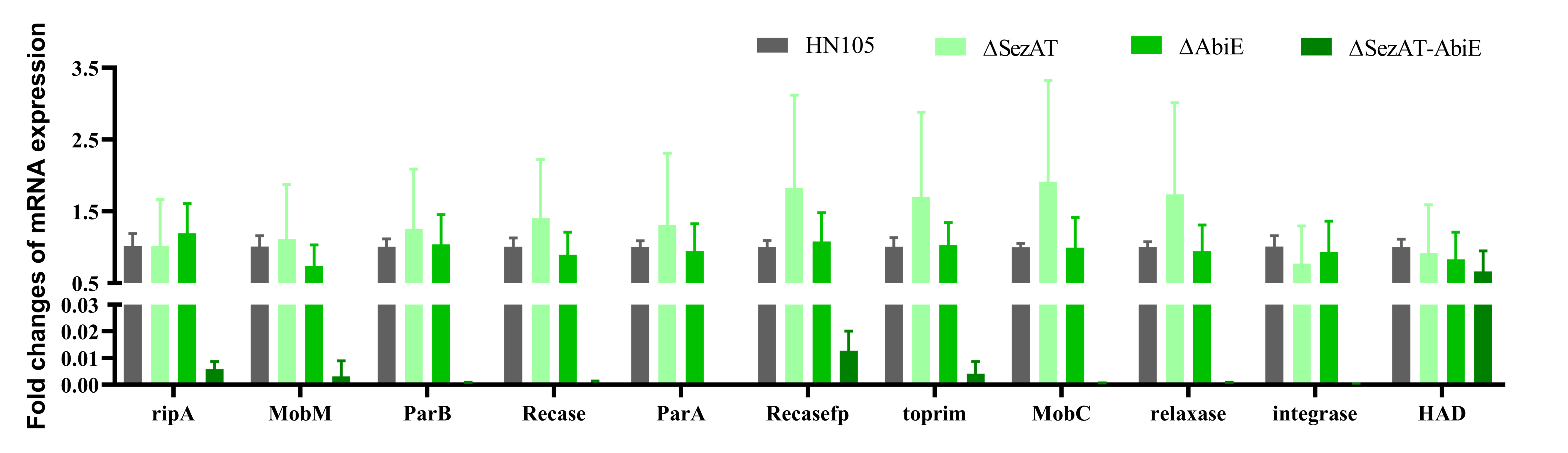

Supplement: S4 Fig — Deletion of SezAT and AbiE does not affect the transcription of excision and integration-related genes. (TIF) [file ppat.1012169.s006.tif]

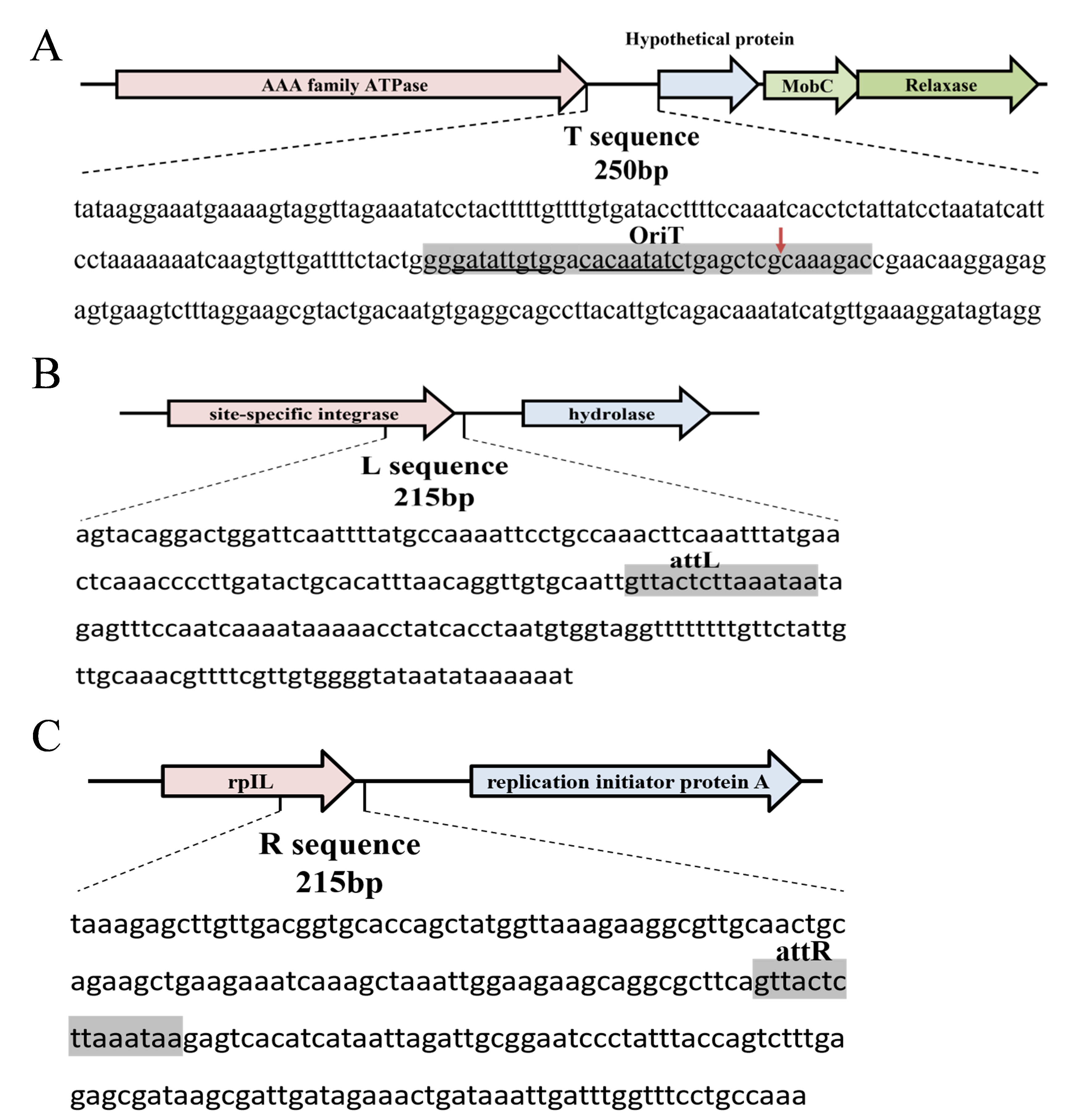

Supplement: S5 Fig — Grey areas represent the sites for oriT, attL, and attR, respectively. Red arrows indicate the predicted recognition and cleavage sites of the relaxase. (TIF) [file ppat.1012169.s007.tif]

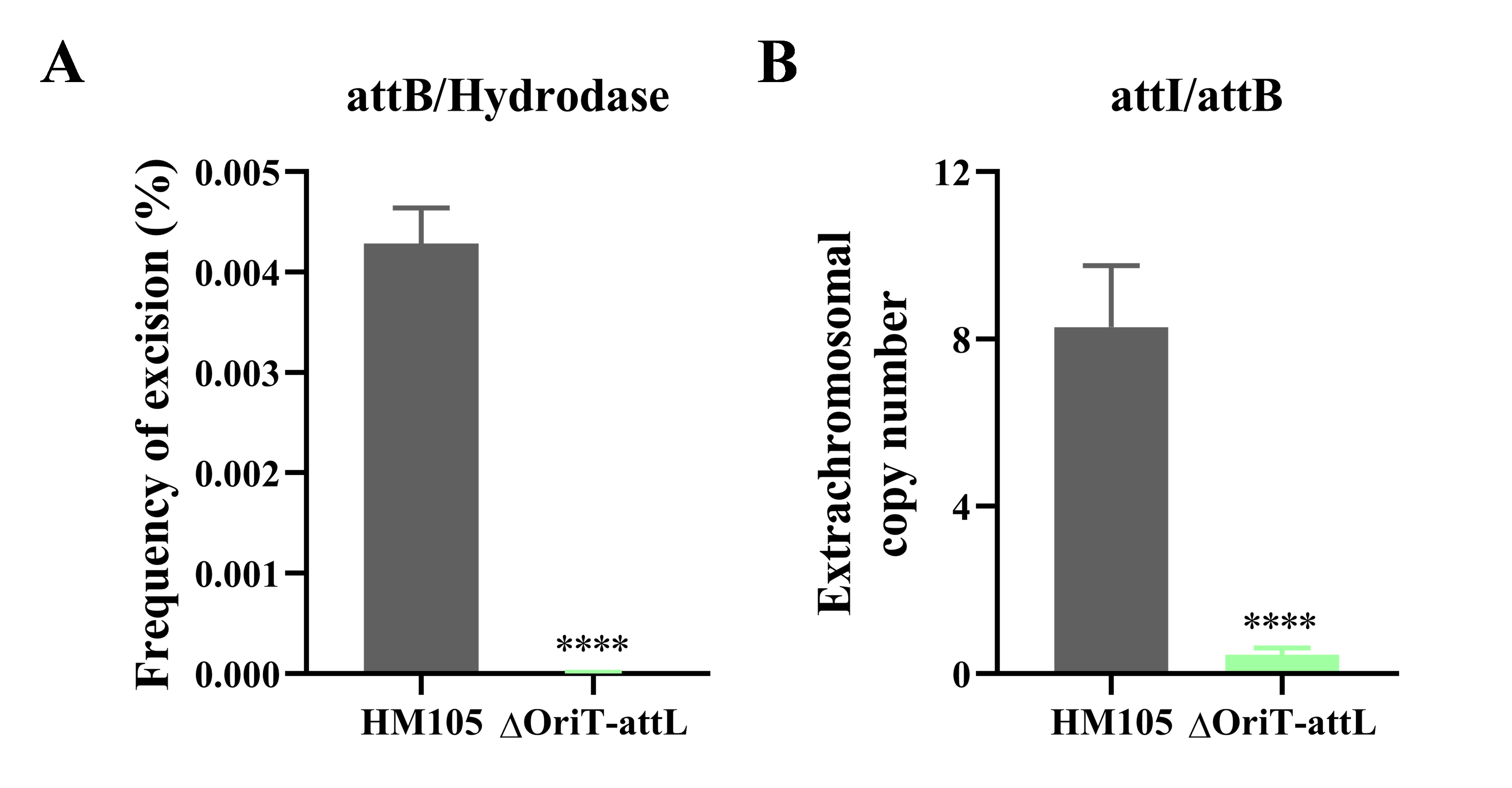

Supplement: S6 Fig — All experiments were conducted independently three times. Unpaired two-tailed Student’s t-test: **** P < 0.0001. (TIF) [file ppat.1012169.s008.tif]

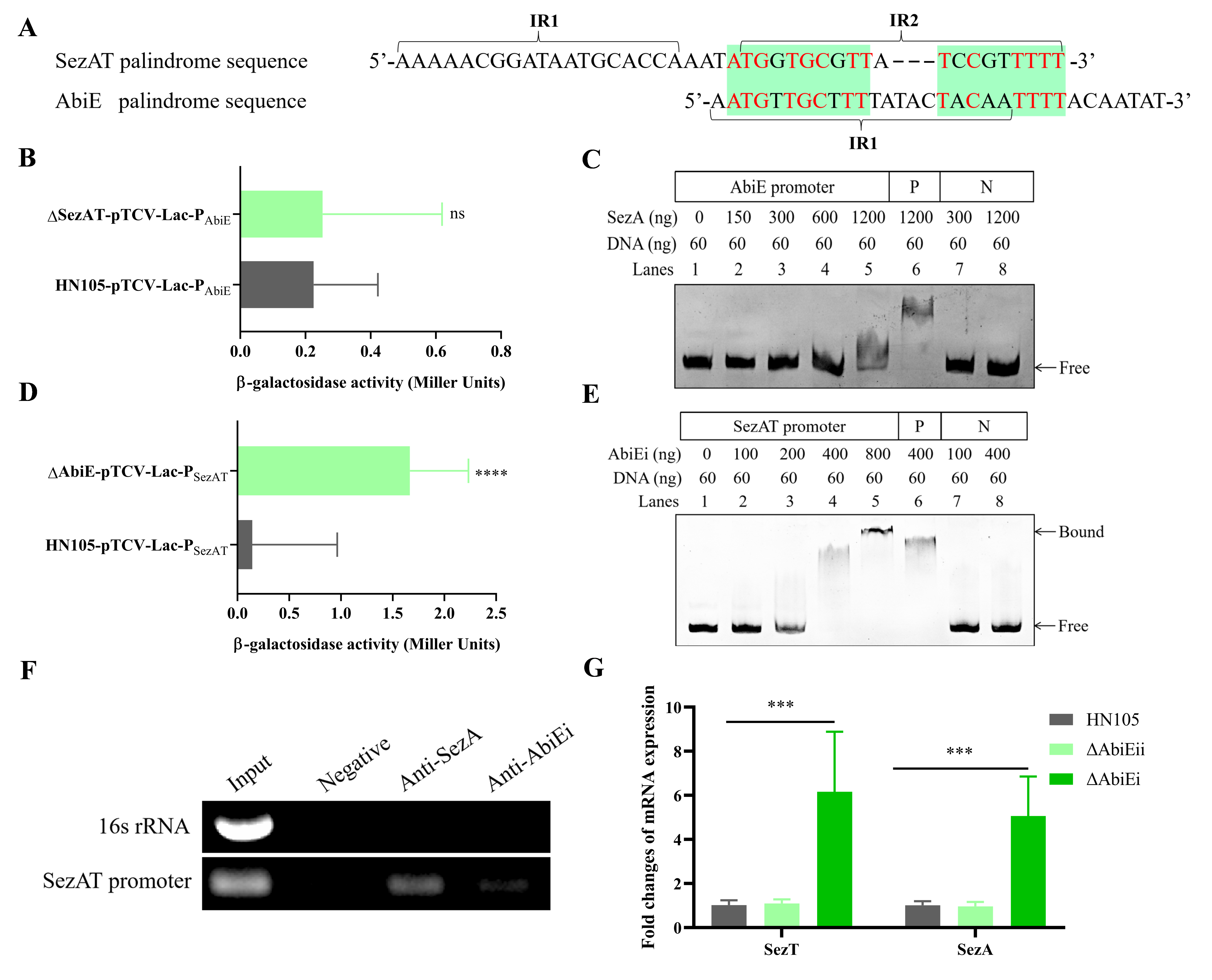

Supplement: S7 Fig — (A) Sequence similarity analysis of AbiE promoter IR1 and IR2 with the SezAT promoter IR1 and IR2. (B) pTCV-lac-PAbiE was integrated to HN105 and ΔSezAT host to determine promoter activity. (C) The EMSA result shown that SezA does not bind to the AbiE promoter. The SezAT promoter served as positive control. (D) pTCV-lac-PSezAT was integrated to HN105 and ΔAbiE host to determine promoter activity. (E) EMSA result shown that AbiEi binds to the SezAT promoter. The AbiE promoter fragment served as positive control. (F) ChIP analysis was performed to detect the binding of AbiEi to SezAT promoter. The Anti-SezA antibody served as a positive control. Normal mouse serum was used as a negative control. The 16S rRNA gene PCR product was used as a negative control. (G) Transcript levels of SezA and SezT in ΔAbiEi and ΔAbiEii. All experiments were conducted independently three times. Unpaired two-tailed Student’s t-test: ns P > 0.05; *** P < 0.001; **** P < 0.0001. (TIF) [file ppat.1012169.s009.tif]

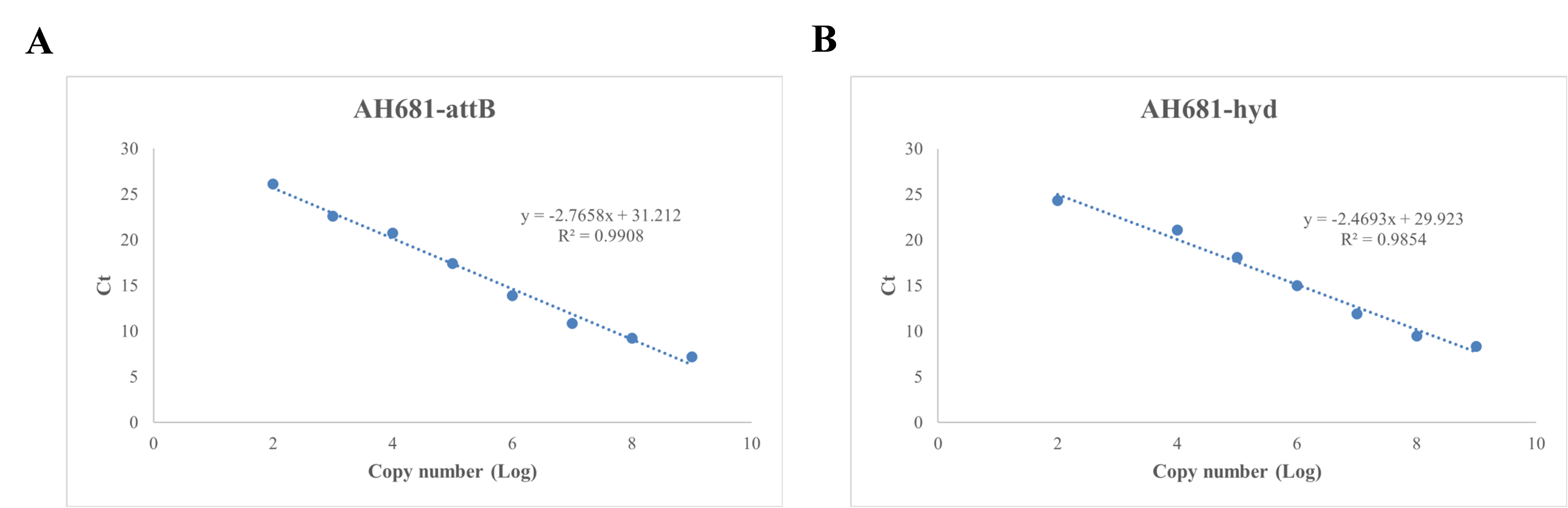

Supplement: S8 Fig — Standard curves for AH681-attB (A) and AH681-hyd (B) copy number analysis. (TIF) [file ppat.1012169.s010.tif]
