# Supplementary material for: Type II and IV toxin-antitoxin systems coordinately stabilize the integrative and conjugative element of the ICESa2603 family conferring multiple drug resistance in Streptococcus suis
Source: PLoS Pathog. 2024 Apr 19;20(4):e1012169. doi: 10.1371/journal.ppat.1012169 (PMC11062541; doi:10.1371/journal.ppat.1012169)
Supplement: S1 Table — (DOCX) [file ppat.1012169.s011.docx]

**S1 Table.** Bacterial strains and plasmids used in this study.

| **Bacterial strains/Plasmids** | **Description^a^** | **Reference** |
| --- | --- | --- |
| **Strains**  HN105 | Serotype 5 wild type | Collected in our lab |
| AH681 | Serotype Chz wild type | Collected in our lab |
| *ΔSezAT* | SezAT deletion mutant in HN105 | This study |
| *ΔSezA* | SezA deletion mutant in HN105 | This study |
| Δ*SezT* | SezT deletion mutant in HN105 | This study |
| Δ*AbiE* | AbiE deletion mutant in HN105 | This study |
| Δ*AbiEi* | AbiEi deletion mutant in HN105 | This study |
| Δ*AbiEii* | AbiEii deletion mutant in HN105 | This study |
| Δ*SezAT-AbiE* | SezAT and AbiE double deletion mutant in HN105 | This study |
| Δ*SezA-AbiEi* | SezA and AbiEi double deletion mutant in HN105 | This study |
| Δ*SezT-AbiEii* | SezT and AbiEii double deletion mutant in HN105 | This study |
| Δ*OriT* | OriT deletion mutant in HN105 | This study |
| Δ*attL* | attL deletion mutant in HN105 | This study |
| ΔOriT-attL | OriT and attL double deletion mutant in HN105 | This study |
| Δ*SHTH* | SHTH deletion mutant in HN105 | This study |
| Δ*AHTH* | AHTH deletion mutant in HN105 | This study |
| AH681-ΔSezAT | SezAT deletion mutant in AH681 | This study |
| AH681-ΔAbiE | AbiE deletion mutant in AH681 | This study |
| AH681-ΔSezAT-AbiE | SezAT and AbiE double deletion mutant in AH681 | This study |
| BTH101 | Reporter strain for Bacterial Two-Hybrid assay | Invitrogen |
| DH5α | Cloning host for maintaining the recombinant plasmids | Invitrogen |
| Top10 | Host for expressing proteins | Invitrogen |
| BL21 (DE3)  **Plasmids** | Host for expressing proteins | Invitrogen |
| pBADHisA | Expression vector; Amp^r^ | Invitrogen |
| pBADHisA-SezT | pBADHisA containing the SezT gene; Amp^r^ | This study |
| pBADHisA-SezAT | pBADHisA containing the SezAT gene; Amp^r^ | This study |
| pBADHisA-AbiEii | pBADHisA containing the AbiEii gene; Amp^r^ | This study |
| pBADHisA-AbiE | pBADHisA containing the AbiE gene; Amp^r^ | This study |
| pBAD33 | Expression vector; Cm^r^ | Invitrogen |
| pBAD33-AbiEii | pBAD33 containing the AbiEii gene; Cm^r^ | This study |
| pBAD33-zeta | pBAD33 containing the zeta gene; Cm^r^ | This study |
| pET28a | Expression vector; Kan^r^ | Invitrogen |
| pET28a-AbiEi | pET28a containing the AbiEi gene; Kan^r^ | This study |
| pET28a-epsilon | pET28a containing the epsilon gene; Kan^r^ | This study |
| pET28a-SUMO | Expression vector; Kan^r^ | Invitrogen |
| pET28a-SUMO-SezA | pET28a-SUMO containing the SezA gene; Kan^r^ | This study |
| pET28a-SUMO-AbiEi | pET28a-SUMO containing the AbiEi gene; Kan^r^ | This study |
| pET28a-SUMO-SezA^Del-SHTH^ | pET28a-SUMO containing the SezADel-SHTH gene; Kan^r^ | This study |
| pET28a-SUMO-AbiEi^Del-AHTH^ | pET28a-SUMO containing the AbiEiDel-AHTH gene; Kan^r^ | This study |
| pCE2-TA/Blunt-zero | Cloning vector; Amp^r^ and Kan^r^ | Invitrogen |
| pCE2-TA/Blunt-zero-attI | pCE2-TA/Blunt-zero containing the attI fragment; Amp^r^ and Kan^r^ | This study |
| pCE2-TA/Blunt-zero-attB | pCE2-TA/Blunt-zero containing the attB fragment; Amp^r^ and Kan^r^ | This study |
| pCE2-TA/Blunt-zero-hydrodase | pCE2-TA/Blunt-zero containing the hydrodase fragment; Amp^r^ and Kan^r^ | This study |
| pTCV-Lac | Gram-positive bacteria-E. coli shuttle vector pTCV-lac; Cm^r^ | [1] |
| pTCV-Lac-P_AbiE_ | pTCV-Lac contains 200bp of AbiE promoter region; Cm^r^ | This study |
| pTCV-Lac-P_SezAT_ | pTCV-Lac contains 200bp of SezAT promoter region; Cm^r^ | This study |
| pTCV-Lac-P_AbiE_-∆IR1 | pTCV-Lac contains 179bp of AbiE-∆IR1 promoter region; Cm^r^ | This study |
| pTCV-Lac-P_AbiE_-∆IR2 | pTCV-Lac contains 179bp of AbiE-∆IR2 promoter region; Cm^r^ | This study |
| pTCV-Lac-P_AbiE_-∆IR1/2 | pTCV-Lac contains 158bp of AbiE-∆IR1/2 promoter region; Cm^r^ | This study |
| pTCV-Lac-P_SezAT_-∆IR1/2 | pTCV-Lac contains 162bp of SezAT-∆IR1/2 promoter region; Cm^r^ | This study |
| pKT25 | Plasmid for Bacterial Two-Hybrid assay; Kan^r^ | Invitrogen |
| pUT18C | Plasmid for Bacterial Two-Hybrid assay; Amp^r^ | Invitrogen |
| pKT25-zip | Control plasmid for Bacterial Two-Hybrid assay; Kan^r^ | Invitrogen |
| pUT18C-zip | Control plasmid for Bacterial Two-Hybrid assay; Amp^r^ | Invitrogen |
| pKT25-AbiEi | pKT25 containing the AbiEi gene; Kan^r^ | This study |
| pUT18C-AbiEii | pUT18C containing the AbiEii gene; Amp^r^ | This study |

**^a^Cm^r^, chloramphenicol resistant; Kan^r^, kanamycin resistant; Amp^r^, ampicillin resistance.**

[1] Poyart C, Trieu-Cuot P. A broad-host-range mobilizable shuttle vector for the construction of transcriptional fusions to beta-galactosidase in gram-positive bacteria. FEMS Microbiol Lett. 1997 Nov 15;156(2):193-8. doi: 10.1111/j.1574-6968.1997.tb12726.x.
