# Supplementary material for: Type II and IV toxin-antitoxin systems coordinately stabilize the integrative and conjugative element of the ICESa2603 family conferring multiple drug resistance in Streptococcus suis
Source: PLoS Pathog. 2024 Apr 19;20(4):e1012169. doi: 10.1371/journal.ppat.1012169 (PMC11062541; doi:10.1371/journal.ppat.1012169)
Supplement: S2 Table — (DOCX) [file ppat.1012169.s012.docx]

**S2 Table.** All primers used in this study.

| Primer | Sequence^a^ |
| --- | --- |
| Construction of Plasmid | |
| pBADHisA-SezT-F | CCGCTCGAGATGAGACTGGAAGAATTT |
| pBADHisA-SezT-R | CCGGAATTCTTATTTATTTTTCTCAAG |
| pBADHisA-SezAT-F | CCGCTCGAGATGATCGGAGACAATATA |
| pBADHisA-SezAT-R | CCGGAATTCTTATTTATTTTTCTCAAG |
| pBADHisA-AbiEii-F | CCCAAGCTTTTAATCAGAATTTATAGAAAT |
| pBADHisA-AbiEii-R | CCGGAATTCATGAATAAAGCTAAGCTAACA |
| pBADHisA-AbiE-F | CCCAAGCTTTTAATCAGAATTTATAGAAATGAG |
| pBADHisA-AbiE-R | CCGGAATTCATGTCAAAAAAAGAGATTCTACTT |
| pBADHisA-F | AGATTAGCGGATCCTACCTG |
| pBADHisA-R | CACTTCTGAGTTCGGCATGG |
| pBAD33-zeta-F | CGAGCTCACAGCGACAATGCTTCAATCT |
| pBAD33-zeta-R | AACTGCAGTTAAAGTCCTGGAAGTTTAGG |
| pBAD33-AbiEii-F | GCCAAAACAGCCAAGCTTTTAATCAGAATTTATAGAAATG |
| pBAD33-AbiEii-R | GCTAGCGAATTCGAGCTCATGAATAAAGCTAAGCTAACAG |
| pBAD33-F | ATGCCATAGCATTTTTATCC |
| pBAD33-R | GATTTAATCTGTATCAGG |
| pET28a-epsilon-F | CGAGCTCAAGCTTTAATTGGTTTAATAA |
| pET28a-epsilon-R | CGCGGATCCATGGCAGTTACTTATGAAAAA |
| pET28a-AbiEi-F | GAGTGCGGCCGCAAGCTTTCATACTAGGACCTCCAGAGTT |
| pET28a-AbiEi-R | GGATCCGAATTCGAGCTCATGTCAAAAAAAGAGATTCTAC |
| pET28a-F | TAATACGACTCACTATAGGG |
| pET28a-R | TGCTAGTTATTGCTCAGCGG |
| HN105-P1 | AAAGTTGGCGTTATCAAAG |
| HN105-P2 | GCCCCATCCTCATCAATCC |
| HN105-P3 | AACAAAGACTCCAGCAGGTGA |
| HN105-P4 | GCGGTCGATAGGAACAACC |
| AH681-P1 | CATCCGTTAATAGGCGAATGA |
| AH681-P2 | CAAACGTAAAGCGCAGCA |
| AH681-P3 | TGATTTGCGGTGATACGTTTC |
| AH681-P4 | TTGACATCTGCTGGCGACA |
| pCE2-TA/Blunt-zero-attI-F | CACATATAGTCATTGGTTTAA |
| pCE2-TA/Blunt-zero-attI-R | CAGGATTTTTGGCAGGAAACC |
| pCE2-TA/Blunt-zero-HN105-attB-F | GTCTTGGCCTTAAAGAAGCTA |
| pCE2-TA/Blunt-zero-HN105-attB-R | TTTAGACAGCATTTTTTATAT |
| pCE2-TA/Blunt-zero-HN105-hydrodase-F | GACCGCGCCAGTTGAAAAATG |
| pCE2-TA/Blunt-zero-HN105-hydrodase-R | CCTTTAATGTCTGTAGACTAG |
| pCE2-TA/Blunt-zero-AH681-attB-F | GACAGCATTTTTTATATTATA |
| pCE2-TA/Blunt-zero-AH681-attB-R | CGTGAAATCACTGGTCTTGGT |
| pCE2-TA/Blunt-zero-AH681-hydrodase-F | GCTACTTGCCCAAAAGTCTCT |
| pCE2-TA/Blunt-zero-AH681-hydrodase-R | ATAAACTAGAGGTCTGGAATC |
| pCE2-TA/Blunt-zero-F | GTAAAACGACGGCCAGT |
| pCE2-TA/Blunt-zero-R | CAGGAAACAGCTATGAC |
| pET28a-SUMO-SezA-F | GGATCCGAATTCGAGCTCATGATCGGAGACAATATAAAA |
| pET28a-SUMO-SezA-R | GAGTGCGGCCGCAAGCTTTTAAGCCACCACCCGATGATG |
| pET28a-SUMO-AbiEi-F | GAGTGCGGCCGCAAGCTTTCATACTAGGACCTCCAGAGTT |
| pET28a-SUMO-AbiEi-R | GGATCCGAATTCGAGCTCATGTCAAAAAAAGAGATTCTAC |
| pET28a-SUMO-SezA^Del-SHTH^-F | CGCGGATCCATGATCGTAGGAGAGGACAAGATGTTA |
| pET28a-SUMO-SezA^Del-SHTH^-R | CCGCTCGAGAGCCACCACCCGATGATGCAC |
| pET28a-SUMO-AbiEi^Del-AHTH^-F | CCGCTCGAGTACTAGGACCTCCAGAGTTTG |
| pET28a-SUMO-AbiEi^Del-AHTH^-R | CGCGGATCCATGTCAAAAAAAACCCAGAATGGAGACTACGAC |
| pTCV-Lac-P_AbiE_-F | CGCGGATCCACTTACTCCTTCCAATGTTGC |
| pTCV-Lac-P_AbiE_-R | CCGGAATTCAGAAAGTGATGATTACATGTT |
| pTCV-Lac-P_AbiE_-∆IR1-F | CGCGGATCCACTTACTCCTTCCTTTTACAATATTTGTAGTGCAA |
| pTCV-Lac-P_AbiE_-∆IR1-R | CCGGAATTCAGAAAGTGATGATTACATGTTT |
| pTCV-Lac-P_AbiE_-∆IR2-F | CGCGGATCCACTTACTCCTTCCAATGTTGCTTTTATACTACAATTTTACAATATTTCTTTTACATTAATGTATTG |
| pTCV-Lac-P_AbiE_-∆IR2-R | CCGGAATTCAGAAAGTGATGATTACATGTT |
| pTCV-Lac-P_AbiE_-∆IR1/2-F | CGCGGATCCACTTACTCCTTCCTTTTACAATATTTCTTTTACATTAATGTATTG |
| pTCV-Lac-P_AbiE_-∆IR1/2-R | CCGGAATTCAGAAAGTGATGATTACATGTT |
| pTCV-Lac-P_SezAT_-F | CCGGAATTCGGGACATTATAACCGATGAGG |
| pTCV-Lac-P_SezAT_-R | CGCGGATCCAGATAAAGACCTCCTCATATT |
| pTCV-Lac-P_SezAT_-∆IR1/2-F | CCGGAATTCGGGACATTATAACCGATGAGG |
| pTCV-Lac-P_lytA_-∆IR1/2-R | CGCGGATCCAGATAAAGACCTCCTCATATTTGCTTTCATTATACCATATATTAGGTTTCTTCGATTGCTTAAA |
| pTCV-Lac-F | GTTGAATAACACTTATTCCTATC |
| pTCV-Lac-R | GAAGTTACTGACGTAAG |
| pKT25-AbiEi-F | ATTCTTAGTTACTTAGGTACCTCATACTAGGACCTCCAGAGT |
| pKT25-AbiEi-R | AGGGTCGACTCTAGAGGATCCCATGTCAAAAAAAGAGATTCTA |
| pKT25-F | TTCGAGTTTCGCGTGAAGGAA |
| pKT25-R | GCTGCGCAACTGTTGGGAAGG |
| pUT18C-AbiEii-F | CTTAGTTATATCGATGAATTCTTAATCAGAATTTATAGAAATGAG |
| pUT18C-AbiEii-R | TCTAGAGGATCCCCGGGTACCGATGAATAAAGCTAAGCTAACAGCA |
| pUT18C-F | GCATACGGCGTGGCGGGGAAA |
| pUT18C-R | TTTCGGTGATGACGGTGAAAA |
| Construction of deletion strains | |
| SezAT-F | GTTTCTTATGTCGATATTGTA |
| SezAT-R | TTTAGAAGATAATAATTGAGC |
| SezAT-U1-F | GTAGCTAGACAAGAAGAAAAG |
| SezAT-U1-R | TTTTCAGCATTATCCAGATAAAGACCTCCTCATATT |
| SezAT-D1-F | GAACCCATCGAATTAATTTATCAAAGAGATAGAACT |
| SezAT-D1-R | AATCATTATCTAATAAAAAAT |
| SacB-CM-F | GGATAATGCTGAAAACTCCTT |
| SacB-CM-R | TAATTCGATGGGTTCCGAGGC |
| R-SezAT-1-F | GTAGCTAGACAAGAAGAAAAG |
| R-SezAT-1-R | AATCATTATCTAATAAAAAAT |
| JC-SacB-CM-F | TAACAAAGCATACTATGGCAA |
| JC-SacB-CM-R | GTGGCTCTAACTTATCCCAAT |
| JC-SezAT-1-F | GACTTATAATTGAAGGAGAAG |
| JC-SezAT-1-R | AGAAATCAGTGTAGAATAAAC |
| SezAT-U2-F | GTAGCTAGACAAGAAGAAAAG |
| SezAT-U2-R | ATCTCTTTGATAAATAGATAAAGACCTCCTCATATT |
| SezAT-D2-F | AGGAGGTCTTTATCTATTTATCAAAGAGATAGAACT |
| SezAT-D2-R | AATCATTATCTAATAAAAAAT |
| R-SezAT-2-F | GTAGCTAGACAAGAAGAAAAG |
| R-SezAT-2-R | AATCATTATCTAATAAAAAAT |
| JC-SezAT-2-F | GATGAAAAGAAAATCCAAAAT |
| JC-SezAT-2-R | CTGTTTGAAGTTTTTTAATAC |
| SezA-F | ATGATCGGAGACAATATAAAA |
| SezA-R | CATCTAAATAGCCATTGTAAC |
| SezA-U1-F | AACGGCAACAATCTAGTCAGT |
| SezA-U1-R | GAGTTTTCAGCATTATCCAGATAAAGACCTCCTCATATT |
| SezA-D1-F | TCGGAACCCATCGAATTACCTTGGATTTTGATGAGTGAT |
| SezA-D1-R | GTAATTCCTTAAGTAGGAGTT |
| R-SezA-1-F | AACGGCAACAATCTAGTCAGT |
| R-SezA-1-R | GTAATTCCTTAAGTAGGAGTT |
| JC-SezA-1-F | GATGTACAGACGCCCGAATCA |
| JC-SezA-1-R | CAGGGATTTGCAGCATATCAT |
| SezA-U2-F | AACGGCAACAATCTAGTCAGT |
| SezA-U2-R | AGATAAAGACCTCCTCATATT |
| SezA-D2-F | ATGAGGAGGTCTTTATCTCCTTGGATTTTGATGAGTGAT |
| SezA-D2-R | GTAATTCCTTAAGTAGGAGTT |
| R-SezA-2-F | AACGGCAACAATCTAGTCAGT |
| R-SezA-2-R | GTAATTCCTTAAGTAGGAGTT |
| JC-SezA-2-F | TAGTCCTATGCGGGCTACCTG |
| JC-SezA-2-R | ATGGTAGCCTTGCGTACTGAG |
| SezT-F | ATGAGACTGGAAGAATTTAGT |
| SezT-R | TTATTTATTTTTCTCAAGTAA |
| SezT-U1-F | GTAGTCCTATGCGGGCTACCT |
| SezT-U1-R | GAGTTTTCAGCATTATCCAGCTTTCGGTTGATCTGGAAT |
| SezT-D1-F | TCGGAACCCATCGAATTAATTTATCAAAGAGATAGAACT |
| SezT-D1-R | AATCATTATCTAATAAAAAAT |
| R-SezT-1-F | GTAGTCCTATGCGGGCTACCT |
| R-SezT-1-R | AATCATTATCTAATAAAAAAT |
| JC-SezT-1-F | GCTTTATAGATACCAGGATAG |
| JC-SezT-1-R | GTAATATGGGAAATGCCGTAT |
| SezT-U2-F | GTAGTCCTATGCGGGCTACCT |
| SezT-U2-R | AGCTTTCGGTTGATCTGGAAT |
| SezT-D2-F | CCAGATCAACCGAAAGCTATTTATCAAAGAGATAGAACT |
| SezT-D2-R | AATCATTATCTAATAAAAAAT |
| R-SezT-2-F | GTAGTCCTATGCGGGCTACCT |
| R-SezT-2-R | AATCATTATCTAATAAAAAAT |
| JC-SezT-2-F | GATCAATACGAAAATTTACCT |
| JC-SezT-2-R | CAAATAATTCATCTTCTGTAC |
| AbiE-F | GAATCTCTCGAGTAGTTCAATAAT |
| AbiE-R | CAGATGAAATTCCTCAATATTTTG |
| AbiE-U1-F | AGGGCTGGCTCTCACTCAATG |
| AbiE-U1-R | GAGTTTTCAGCATTATCCTTGTGACAAAAAAATAAAAAG |
| AbiE-D1-F | TCGGAACCCATCGAATTAGTATAGAATCCCTTCTTTTTCTAG |
| AbiE-D1-R | AGCATTTATTTCTTTACCAGTTTG |
| R-AbiE-1-F | AGGGCTGGCTCTCACTCAATG |
| R-AbiE-1-R | AGCATTTATTTCTTTACCAGT |
| JC-AbiE-1-F | GACCTTTTTGAAAACTCTATT |
| JC-AbiE-1-R | GAGATATTTTTAATTGTGGAC |
| AbiE-U2-F | AGGGCTGGCTCTCACTCAATG |
| AbiE-U2-R | AAAGAAGGGATTCTATACTTGTGACAAAAAAATAAAAAG |
| AbiE-D2-F | GTATAGAATCCCTTCTTTTTCTAG |
| AbiE-D2-R | AGCATTTATTTCTTTACCAGTTTG |
| R-AbiE-2-F | AGGGCTGGCTCTCACTCAATG |
| R-AbiE-2-R | AGCATTTATTTCTTTACCAGT |
| JC-AbiE-2-F | GGGCAAGTGAGTGGTATT |
| JC-AbiE-2-R | TTCAGTCGCAGATGTCGTATT |
| AbiEi-F | GTCGCATATTCATAGAGTTTT |
| AbiEi-R | CTTGAATTTATAGAAAACCAC |
| AbiEi-U1-F | TTTTTGAATAATTTTGCCATTG |
| AbiEi-U1-R | GAGTTTTCAGCATTATCCCATCGAGAAAAAATAGATACTG |
| AbiEi-D1-F | TCGGAACCCATCGAATTAACTTACTCCTTCCAATGTTGCT |
| AbiEi-D1-R | AGCAGTTTTGCCATAGTTTATG |
| R-AbiEi-1-F | TTTTTGAATAATTTTGCCATT |
| R-AbiEi-1-R | AGCAGTTTTGCCATAGTTTAT |
| JC-AbiEi-1-F | GTATCTTCTGAAAGTGTTATT |
| JC-AbiEi-1-R | ATTTTTAATTGTGGACGAATC |
| AbiEi-U2-F | TTTTTGAATAATTTTGCCATTG |
| AbiEi-U2-R | CATCGAGAAAAAATAGATACTG |
| AbiEi-D2-F | ATCTATTTTTTCTCGATGACTTACTCCTTCCAATGTTGCT |
| AbiEi-D2-R | AGCAGTTTTGCCATAGTTTATG |
| R-AbiEi-2-F | TTTTTGAATAATTTTGCCATT |
| R-AbiEi-2-R | AGCAGTTTTGCCATAGTTTAT |
| JC-AbiEi-2-F | TCATCACGGAATTAAAGGTTA |
| JC-AbiEi-2-R | TTCAGTCGCAGATGTCGTATT |
| AbiEii-F | TTAATCAGAATTTATAGAAATG |
| AbiEii-R | ATGAATAAAGCTAAGCTAACAG |
| AbiEii-U1-F | TGGGATGATGAGGGCTGGCTC |
| AbiEii-U1-R | GAGTTTTCAGCATTATCCTATAGCTATACAAAGGGGATT |
| AbiEii-D1-F | TCGGAACCCATCGAATTAATCCTCCTTTGAATATATAGT |
| AbiEii-D1-R | AAGTACGGTATTTTTTACTCT |
| R-AbiEii-1-F | TGGGATGATGAGGGCTGGCTC |
| R-AbiEii-1-R | AAGTACGGTATTTTTTACTCT |
| JC-AbiEii-1-F | GTAAATTATCTGGAGTGGATA |
| JC-AbiEii-1-R | GTGGTAGCCGTGATAGTTTGC |
| AbiEii-U2-F | TGGGATGATGAGGGCTGGCTC |
| AbiEii-U2-R | TATAGCTATACAAAGGGGATT |
| AbiEii-D2-F | CCCCTTTGTATAGCTATAATCCTCCTTTGAATATATAGT |
| AbiEii-D2-R | AAGTACGGTATTTTTTACTCT |
| R-AbiEii-2-F | TGGGATGATGAGGGCTGGCTC |
| R-AbiEii-2-R | AAGTACGGTATTTTTTACTCT |
| JC-AbiEii-2-F | CTCCTTCCTTATCTCCGAAAC |
| JC-AbiEii-2-R | CACTTCAACATTACGGAAACT |
| JC-OriT-F | GTTAGTGTATTAAGTTTGGG |
| JC-OriT-R | GCGAGCTCAGATATTGTGTC |
| OriT-U1-F | AACTCTCAATTTCTCATTTG |
| OriT-U1-R | GAGTTTTCAGCATTATCCCAGTAGAAAATCAACACTTG |
| OriT-D1-F | TCGGAACCCATCGAATTACGAACAAGGAGAGAGTGAAGT |
| OriT-D1-R | CTGTTTTACCGGGATTGAGGA |
| R-OriT-1-F | AACTCTCAATTTCTCATTTG |
| R-OriT-1-R | CTGTTTTACCGGGATTGAGG |
| JC-OriT-1-F | GAATCACAGGGATATGTTAGT |
| JC-OriT-1-R | ATTTTTAATTGTGGACGAATC |
| OriT-U2-F | AACTCTCAATTTCTCATTTG |
| OriT-U2-R | CAGTAGAAAATCAACACTTG |
| OriT-D2-F | AGTGTTGATTTTCTACTGCGAACAAGGAGAGAGTGAAGT |
| OriT-D2-R | CTGTTTTACCGGGATTGAGGA |
| R-OriT-2-F | AACTCTCAATTTCTCATTTG |
| R-OriT-2-R | CTGTTTTACCGGGATTGAGG |
| JC-OriT-2-F | ATAATTTGGATAACCGCTAT |
| JC-OriT-2-R | TCTTGCGACAACTAGAACTT |
| JC-attL-F | GAACGACTTATTGAATTGGAT |
| JC-attL-R | TTATTTAAGAGTAACAATTGC |
| attL-U1-F | CAAAGCCATTAAAAGCAATA |
| attL-U1-R | GAGTTTTCAGCATTATCCAATTGCACAACCTGTTAAAT |
| attL-D1-F | TCGGAACCCATCGAATTATAGAGTTTCCAATCAAAATA |
| attL-D1-R | TATTCTTTACTGTTCTATCG |
| R-attL-you-F | CAAAGCCATTAAAAGCAATA |
| R-attL-you-R | TATTCTTTACTGTTCTATCG |
| JC-attL-1-F | GTCTTAGACATAGTCATGCT |
| JC-attL-1-R | TTTGCTGTTCAGGGATTTGC |
| attL-U2-F | CAAAGCCATTAAAAGCAATA |
| attL-U2-R | AATTGCACAACCTGTTAAAT |
| attL-D2-F | ACAGGTTGTGCAATTTAGAGTTTCCAATCAAAATA |
| attL-D2-R | TATTCTTTACTGTTCTATCG |
| R-attL-2-F | CAAAGCCATTAAAAGCAATA |
| R-attL-2-R | TATTCTTTACTGTTCTATCG |
| JC-attL-2-F | TTTATATGTGGCAAGACGAA |
| JC-attL-2-R | AGTCGGCTTATTGGTACTTT |
| JC-SezA-HTH-F | GACAATATAAAATCACTACGC |
| JC-SezA-HTH-R | CTTTGATAAAATGGCTGCTCC |
| SezA-HTH-U1-F | AACAATCTAGTCAGTTGTCAT |
| SezA-HTH-U1-R | TTTTCAGCATTATCCGATCATAGATAAAGACCTCCT |
| SezA-HTH-D1-F | GAACCCATCGAATTAGTAGGAGAGGACAAGATGTTA |
| SezA-HTH-D1-R | TTTGAATCTGGTCAAAGAGAT |
| R-SezA-HTH-1-F | AACAATCTAGTCAGTTGTCAT |
| R-SezA-HTH-1-R | TTTGAATCTGGTCAAAGAGAT |
| JC-SezA-HTH-1-F | TAGTCCTATGCGGGCTACCTG |
| JC-SezA-HTH-1-R | CCGGCTAATGCAAAGACGATG |
| SezA-HTH-U2-F | AACAATCTAGTCAGTTGTCAT |
| SezA-HTH-U2-R | CTTGTCCTCTCCTACGATCATAGATAAAGACCTCCT |
| SezA-HTH-D2-F | TCTTTATCTATGATCGTAGGAGAGGACAAGATGTTA |
| SezA-HTH-D2-R | TTTGAATCTGGTCAAAGAGAT |
| R-SezA-HTH-2-F | AACAATCTAGTCAGTTGTCAT |
| R-SezA-HTH-2-R | TTTGAATCTGGTCAAAGAGAT |
| JC-SezA-HTH-2-F | AGTTGGAGAAGCGAATAGAGG |
| JC-SezA-HTH-2-R | TTCGGTTGATCTGGAATCGTC |
| JC-AbiEi-HTH-F | GTTACGTCAAAATATTGAGGA |
| JC-AbiEi-HTH-R | TACTTGAATTTATAGAAAACC |
| AbiEi-HTH-U1-F | CATCAAAAATAGCTTTATAGT |
| AbiEi-HTH-U1-R | TTTTCAGCATTATCCACCCAGAATGGAGACTACGAC |
| AbiEi-HTH-D1-F | GAACCCATCGAATTATTTTTTTGACATACTTACTCC |
| AbiEi-HTH-D1-R | ATAGTTTATGTAGTCAGATAC |
| R-AbiEi-HTH-1-F | CATCAAAAATAGCTTTATAGT |
| R-AbiEi-HTH-1-R | ATAGTTTATGTAGTCAGATAC |
| JC-AbiEi-HTH-1-F | GATGAATCACAAAATCACAGA |
| JC-AbiEi-HTH-1-R | TTCAGGGATTTGCAGCATATC |
| AbiEi-HTH-U2-F | CATCAAAAATAGCTTTATAGT |
| AbiEi-HTH-U2-R | AGTATGTCAAAAAAAACCCAGAATGGAGACTACGAC |
| AbiEi-HTH-D2-F | GTCTCCATTCTGGGTTTTTTTTGACATACTTACTCC |
| AbiEi-HTH-D2-R | ATAGTTTATGTAGTCAGATAC |
| R-AbiEi-HTH-2-F | CATCAAAAATAGCTTTATAGT |
| R-AbiEi-HTH-2-R | ATAGTTTATGTAGTCAGATAC |
| JC-AbiEi-HTH-2-F | TTGTCGCATATTCATAGAGTT |
| JC-AbiEi-HTH-2-R | ATATCATGCGGTACGGTGACA |
| AH681-SezAT-F | TCTGAATGCGGTCAAAGATCT |
| AH681-SezAT-R | ATGATTGGAGACAATATTAAA |
| AH681-SezAT-U1-F | ATTCATCCGGTCCTTTTTTAT |
| AH681-SezAT-U1-R | TTTTCAGCATTATCCTTTATCAAAGAGACAGGACTT |
| AH681-SezAT-D1-F | GAACCCATCGAATTAAGATAAAGACCTCCTCATATT |
| AH681-SezAT-D1-R | AAACGGCAACAATCTAGTCAG |
| R-AH681-SezAT-1-F | ATTCATCCGGTCCTTTTTTAT |
| R-AH681-SezAT-1-R | AAACGGCAACAATCTAGTCAG |
| JC-AH681-SezAT-1-F | CTCCTGTAATCGTTCCCGTCC |
| JC-AH681-SezAT-1-R | GTGTTTGTTTGCCGTAATGTT |
| AH681-SezAT-U2-F | ATTCATCCGGTCCTTTTTTAT |
| AH681-SezAT-U2-R | TTTATCAAAGAGACAGGACTT |
| AH681-SezAT-D2-F | TGTCTCTTTGATAAAAGATAAAGACCTCCTCATATT |
| AH681-SezAT-D2-R | AAACGGCAACAATCTAGTCAG |
| R-AH681-SezAT-2-F | ATTCATCCGGTCCTTTTTTAT |
| R-AH681-SezAT-2-R | AAACGGCAACAATCTAGTCAG |
| JC-AH681-SezAT-2-F | AATGGCATTGTTTCTATAACT |
| JC-AH681-SezAT-2-R | GTAGTCCTATGCGGGCTACCT |
| AH681-AbiE-F | GCAAAGCAGTATTCAATATGT |
| AH681-AbiE-R | ATGGATTTGTTAGAGAAACCC |
| AH681-AbiE-U1-F | CTTGATTATTTGACAATCGAA |
| AH681-AbiE-U1-R | TTTTCAGCATTATCCACAATACTGCTAAAAAAAGAG |
| AH681-AbiE-D1-F | GAACCCATCGAATTAACCACTACCTCCAAAACAATA |
| AH681-AbiE-D1-R | AACGTGGTTTAGGCTTAGGAC |
| R-AH681-AbiE-1-F | CTTGATTATTTGACAATCGAA |
| R-AH681-AbiE-1-R | AACGTGGTTTAGGCTTAGGAC |
| JC-AH681-AbiE-1-F | TGTTTCATTTGCCCGTCCATA |
| JC-AH681-AbiE-1-R | GATGTGGTAGCCGTGATAGTT |
| AH681-AbiE-U2-F | CTTGATTATTTGACAATCGAA |
| AH681-AbiE-U2-R | ACAATACTGCTAAAAAAAGAG |
| AH681-AbiE-D2-F | TTTTAGCAGTATTGTACCACTACCTCCAAAACAATA |
| AH681-AbiE-D2-R | AACGTGGTTTAGGCTTAGGAC |
| R-AH681-AbiE-2-F | CTTGATTATTTGACAATCGAA |
| R-AH681-AbiE-2-R | AACGTGGTTTAGGCTTAGGAC |
| JC-AH681-AbiE-2-F | TCTTTGGGAATCAAGGATAAT |
| JC-AH681-AbiE-2-R | GGTTACGCTTACGGCCAATGT |
| qRT-PCR | |
| Q-SezA-F | AACGTGGAGCAGCCATTT |
| Q-SezA-R | GGCCAAGTCATCACTCATCAA |
| Q-SezT-F | CGTGGACTATACCAAGGGATTT |
| Q-SezT-R | GTCTGACGAGGAACTTGAGTAG |
| Q-AbiEi-F | GCAGGTAAAGCGCTGAGATA |
| Q-AbiEi-R | TCCTAACCCAGAATGGAGACTA |
| Q-AbiEii-F | GTAATCGGCTGAGGAGTTACAA |
| Q-AbiEii-R | GTGATGACTATGGTGGCTATCG |
| Q-attI-F | AACAGGTTGTGCAATTGTTACTC |
| Q-attI-R | TCGCTTATCGCTCTCAAAGAC |
| Q-attB-F | GCAGAAGCTGAAGAAATCAAAG |
| Q-attB-R | ACCACATTAGGTGATAGGTTT |
| Q-hydrodase-F | CTCGCGGAAACAGTGAGTTAT |
| Q-hydrodase-R | AGTCGGCTTATTGGTACTTTGG |
| Q-AH681-attB-F | ACCACATCAGGTGATAGGTTT |
| Q-AH681-attB-R | CTGCAGAAGCTGAAGAAATCAA |
| Q-AH681-hydrodase-F | CATTTGGTGCATCCGTTAATAGG |
| Q-AH681-hydrodase-R | GAGATCTCTCGCGGAAACAG |
| Q-ripA-F | CTGATGGCACTATTAGGCTGTT |
| Q-ripA-R | TCACTAGAGGACTGTTGGACTT |
| Q-MobM-F | AGCGTGATGATGGTGGAAAC |
| Q-MobM-R | GTCAAGAGCCGTCTGGAAATAC |
| Q-ParB-F | TAATCACACGCCCGAAAGAG |
| Q-ParB-R | AACGCAGGCACGGTATTTA |
| Q-Recase-F | GGCATTTATTGCCGCTTATCT |
| Q-Recase-R | TCCGTGAGGATGGATTTCTG |
| Q-ParA-F | TGTGCTGTCCGTATCAACTAAA |
| Q-ParA-R | TCCCTTTACAAGCAGAAGAAGAA |
| Q-Recasefp-F | TCTAAGCTGCCCGAAGAATG |
| Q-Recasefp-R | GTGGAAGCCTACGACTTGTAATA |
| Q-toprim-F | GATAACGATGAGGCAGGAAGAG |
| Q-toprim-R | GTCCTTGCAATGGTGGTAAATC |
| Q-MobC-F | TGTCACCATTAACACGGATACC |
| Q-MobC-R | CGCACGCGCAATTTGATTA |
| Q-relaxase-F | CCGTGAGACAACCTCATACTTT |
| Q-relaxase-R | GTCGGTCGTCTGTATGGTATTG |
| Q-integrase-F | GACGGCTCTTCCTCATCATAAA |
| Q-integrase-R | CCTCGCAATCTCTAGGAGTTATT |
| Q-HAD-F | CTCGCGGAAACAGTGAGTTAT |
| Q-HAD-R | AGTCGGCTTATTGGTACTTTGG |
| parC-F | CTGCTCCTCGCTAAACTCATAG |
| parC-R | GAAACGCCTCCATATCGTAGAA |
| ChIP | |
| 16s rRNA-F | GCTTAACCATAGTACGCTTTG |
| 16s rRNA-R | TCATCGTTTACGGCGTGGACT |
| T-F | TATAAGGAAATGAAAAGTAGGT |
| T-R | CCTACTATCCTTTCAACATGAT |
| L-F | AGTACAGGACTGGATTCAATTT |
| L-R | ATTTTTTATATTATACCCCACA |
| R-F | TAAAGAGCTTGTTGACGGTGC |
| R-R | TTTGGCAGGAAACCAAATCAA |
| EMSA | |
| EMSA-SezAT-F | GGGACATTATAACCGATGAGG |
| EMSA-SezAT-R | AGATAAAGACCTCCTCATATT |
| EMSA-SezAT-∆IR1/IR2-F | GGGACATTATAACCGATGAGG |
| EMSA-SezAT-∆IR1/IR2-R | AGATAAAGACCTCCTCATATTTGCTTTCATTATACCATATATTAGGTTTCTTCGATTGCTTAAA |
| EMSA-AbiE-F | ACTTACTCCTTCCAATGTTGC |
| EMSA-AbiE-R | AGAAAGTGATGATTACATGTT |
| EMSA-AbiE-∆IR1-F | ACTTACTCCTTCCTTTTACAATATTTGTAGTGCAA |
| EMSA-AbiE-∆IR1-R | AGAAAGTGATGATTACATGTTT |
| EMSA-AbiE-∆IR2-F | ACTTACTCCTTCCAATGTTGCTTTTATACTACAATTTTACAATATTTCTTTTACATTAATGTATTG |
| EMSA-AbiE-∆IR2-R | AGAAAGTGATGATTACATGTT |
| EMSA-AbiE-∆IR1/2-F | ACTTACTCCTTCCTTTTACAATATTTCTTTTACATTAATGTATTG |
| EMSA-AbiE-∆IR1/2-R | AGAAAGTGATGATTACATGTT |
| EMSA-T-F | TATAAGGAAATGAAAAGTAGGT |
| EMSA-T-R | CCTACTATCCTTTCAACATGAT |
| EMSA-T-∆oriT-F1 | TATAAGGAAATGAAAAGTAG |
| EMSA-T-∆oriT-R1 | CTCTCTCCTTGTTCGCAGTAGAAAATCAACACTTG |
| EMSA-T-∆oriT-F2 | GTTGATTTTCTACTGCGAACAAGGAGAGAGTGAAGT |
| EMSA-T-∆oriT-R2 | CCTACTATCCTTTCAACATGA |
| EMSA-L-F | AGTACAGGACTGGATTCAATTT |
| EMSA-L-R | ATTTTTTATATTATACCCCACA |
| EMSA-L-∆attL-F1 | AGTACAGGACTGGATTCAATTT |
| EMSA-L-∆attL-R1 | TGATTGGAAACTCTAAATTGCACAACCTGTTAAATGT |
| EMSA-L-∆attL-F2 | ACAGGTTGTGCAATTTAGAGTTTCCAATCAAAATAAA |
| EMSA-L-∆attL-R2 | ATTTTTTATATTATACCCCACA |
| EMSA-16s-F | GCTTAACCATAGTACGCTTTG |
| EMSA-16s-R | TCATCGTTTACGGCGTGGACT |

^a^ Underlined nucleotides denote restriction enzyme sites.
