# Supplementary material for: Type II and IV toxin-antitoxin systems coordinately stabilize the integrative and conjugative element of the ICESa2603 family conferring multiple drug resistance in Streptococcus suis
Source: PLoS Pathog. 2024 Apr 19;20(4):e1012169. doi: 10.1371/journal.ppat.1012169 (PMC11062541; doi:10.1371/journal.ppat.1012169)
Supplement: Attachment — Submitted filename: Response to reviews.doc [file ppat.1012169.s013.doc]

**Responses to reviewers’ comments** (Manuscript ID: PPATHOGENS-D-24-00118)

Dear editor: We sincerely thank the editor and reviewers for the constructive criticisms and valuable comments, which were of great help in revising the manuscript. We have carefully considered the comments and revised the manuscript accordingly. With these improvements, we hope that the current version can meet the Journal’s standards for publication. The following is a point-by-point list of responses to all comments and a list of changes we have made in the manuscript.

***Response to Reviewer 1 Comments***

Reviewer #1: The manuscript is well presented in general. The amount of work is overwhelming, and very interesting results are convincing and well discussed. I have only a few comments that I would like the authors to consider.

Thanks for your thoughtful comments. We have revised our manuscript based on the

following comments.

Minor Issues: Reviewer #1: GENERAL:

1. I would recommend being more specific in the title: type of TA, bacterial species, and type of ICE. As it is now, it has little information.

Thanks for your kindly comment. We have modified the title to “Type Ⅱ and Ⅳ toxin-antitoxin systems coordinately stabilize the integrative and conjugative element of the ICE*Sa*2603 family conferring multiple drug resistance in *Streptococcus suis*”.

2. Although in general the English is correct, it needs editing throughout.

Thanks for your comment. We have revised the language throughout the text.

SPECIFIC:

3. Lines 140 and around it: a recent review on the hypothesis and roles of type II TAs, with a focus focusing on PezAT should be be cited (https://doi.org/10.1093/femsre/fuad052).

Thanks for your comment. We have included this reference in the resubmitted manuscript.

4. Line 172: &ldquo;Numerous studies&hellip;&rdquo; but they cite just two papers. Correct this or cite at least 3-5 more papers.

Thanks for your comment. We have added references in the resubmitted manuscript.

5. Line 251 and following: In many cases, the antitoxin binds poorly to its DNA target, needing the toxin as a co-repressor, which does not seem to be the case here. It would be interesting if the authors discuss this.

Numerous studies have demonstrated that antitoxins can effectively bind to DNA targets to achieve negative regulatory effects, such as the type Ⅱ antitoxin MqsA in *Escherichia coli* (PMID: 21516113), the antitoxin PezA in *Streptococcus pneumoniae* (PMID: 17488720), and the type Ⅳ antitoxin AbiEi in *Streptococcus agalactiae* (PMID: 24465005) and *Mycobacterium tuberculosis* (PMID: 32519742). Although it has been shown that in *Staphylococcus aureus*, the formation of a complex between the toxin SavS and the antitoxin SavR enhances antitoxin binding to the promoter (PMID: 29440365), it has also been shown that the formation of a complex between the toxin HigB and the antitoxin HigA in *Pseudomonas aeruginosa* prevents the antitoxin DNA-binding process (PMID: 30882983), suggesting that type Ⅱ toxins may or may not act as co-repressors. In the present study, further exploration is needed on the effect of the type II toxin SezT on DNA-binding capacity after forming a complex with the antitoxin SezA. On the other hand, the type IV toxin AbiEii was unable to interact with the antitoxin AbiEi. Consequently, the toxin AbiEii could not directly influence the DNA-binding domain of the antitoxin and affect its binding capacity. We have discussed this in the Discussion section.

6. Discussion: lines 440-450: Cite the work on ICESag-rpsl, which encodes a functional relaxase involved in the mobilization of the element and also in plasmid mobilization (<https://doi.org/10.1098/rsob.160084>)

Thanks for your comment. We have included this reference in the resubmitted manuscript.

***Response to Reviewer 2 Comments***

Reviewer #2: This manuscript investigates how two antitoxins, SezA and AbiEi, promote the stability of an integrative and conjugative element (ICE) in the genome of *Streptococcus suis*. The antitoxins, rather than the toxins, were found to be important for maintaining the genetic stability of the ICE. AbiEi was found to interact with the oriT, and SezA was found to bind in the attL site. This interaction likely impedes the excision and subsequent loss of the ICE, highlighting a novel mechanism of ICE stabilization mediated by antitoxins. The results are exciting. However, there are a few issues that lessen the impact of the work.

Thanks for your thoughtful comments. We have revised our manuscript based on the

following comments.

Major Issues: Reviewer #2: The authors propose an intriguing interaction between AbiEi-oriT and SezA-AttL contributing to the stabilization of ICE elements. However, the evidence presented does not fully substantiate this hypothesis. To bolster their claims, the authors should consider generating deletion mutants for the DNA-binding domains of AbiEi and SezA. This would provide crucial insights into the role of antitoxin DNA-binding in ICE stability. Furthermore, a double deletion mutant involving oriT and attL, in the genetic background of ∆SezA-AbiEi mutants, would be invaluable in supporting the hypothesis if the ICE stability could be restored.

We appreciate these valuable comments very much. To determine the role of antitoxin DNA-binding in ICE stability, we further constructed deletion mutants of the DNA-binding domain, ∆*SHTH* and ∆*AHTH* (Figure 5A and B). By determining the excision frequency and extrachromosomal copy number of ICE*Ssu*HN105, it was found that the excision frequency of ICE was significantly increased, and the extrachromosomal copy number was significantly decreased in ∆*SHTH* and ∆*AHTH* (Figure 5C and D). The results were consistent with antitoxin deletion and deletion of the TA system, suggesting that the DNA-binding domain of the antitoxin play an important role in the stabilization of ICE. Furthermore, we expressed and purified the proteins SezA^Del-SHTH^ and AbiEi^Del-AHTH^, which lack the DNA-binding domain. It was confirmed by EMSA that after the loss of the DNA-binding domain of the antitoxin, SezA^Del-SHTH^ was unable to bind to its own promoter and L sequence (Figure 5E and F), and AbiEi^Del-AHTH^ was also unable to bind to its own promoter and T sequence (Figure 5G and H). These results suggest that the DNA-binding domain of the antitoxin plays a crucial role in self-regulation and ICE stabilization. Please see Figure 5 and the figure legend in the resubmitted manuscript for more details.

Since ICE*Ssu*HN105 has been lost after the double deletion of SezA and AbiEi, no further deletion mutations can be constructed to *OriT* and *attL* on ICE. To further investigate the role of *OriT* and *attL* in ICE stabilization, we constructed a double deletion strain of *OriT* and *attL*. By determining the excision frequency and extrachromosomal copy number of ICE in double deletion, ICE could not be excised, and the extrachromosomal copy number was close to zero. These results suggest that *OriT* and *attL* are required for the stabilization of ICE*Ssu*HN05. Please see Supplementary Figure S6 in the resubmitted manuscript.

What is the extrachromosomal copy number of the attL deletion mutant?

We further determined the extrachromosomal copy number of ICE in the ∆*attL* and observed a significant decrease to almost zero after the deletion of *attL*. The combined excision frequency results indicate that ICE could hardly be excised from the chromosome after the deletion of the *attL* site, and thus the extrachromosomal copy number was close to zero. These results suggest that the *attL* site plays a crucial role in stabilizing the ICE. We have included this result in the resubmitted manuscript. Please see Figure 4K.

Minor Issues: Reviewer #2: In Figures 3H and 3I, the omission of protein concentrations leaves a gap in understanding the experimental setup, especially in estimating the dissociation constant. The inclusion of these details would significantly enhance the interpretability of the results.

Thanks for your kindly comment.

The concentrations of AbiEi in Figure 3H are 0 (0 ng), 289.86 (200 ng), 579.71 (400 ng), 869.57 (600 ng), 1159.4 (800 ng), 1159.4 (800 ng), 289.86 (200 ng) and 869.57 nM (600 ng) in that order.

The concentrations of AbiEi in Figure 3I are 0 (0 ng), 144.93 (100 ng), 289.86 (200 ng), 579.71 (400 ng), 1159.4 (800 ng), 1159.4 (800 ng), 144.93 (100 ng) and 579.71 nM (400 ng).

The AbiEi concentrations used to assess the dissociation constant in Figure 3J are 0 (0 ng), 72.46 (50 ng), 144.93 (100 ng), 289.86 (200 ng), 579.71 (400 ng), 1159.4 (800 ng) and 2318.8 nM (1600 ng).

We have included these details in the resubmitted manuscript. Please see the legends for Figures 3 and 4.

The Kd values mentioned on lines 288 and 315 warrant a second look, as transcription factors are known to bind DNA with nanomolar affinity. This discrepancy suggests a possible miscalculation or misinterpretation that should be addressed to ensure the accuracy of the findings.

We are very sorry that the *K*_D_ values of 0.0044 nM and 0.068 nM were miscalculated. We have replaced them with the values of 153.07 nM and 1955.59 nM in the resubmitted manuscript, respectively. Please see lines 294 and 322 in the Results section, and the resubmitted Figures 3J and 4G.

Line 82, please provide the full name of SXT.

Thanks for your comment. We have revised it to “ICE SXT (ICE carrying sulfamethoxazole and trimethoprim resistance)” as your suggestion.

All of the figures&#39; resolutions are too low.

Thanks for your kindly comment. The PDF file is automatically generated by the review system, and the resolutions of the figures in the PDF are relatively low. Above the PDF, there is a link to open the original figure, which is very clear. We have re-uploaded the figures in the manuscript.

***Response to Reviewer 3 Comments***

Reviewer #3: Upon reviewing the manuscript focused on the study of toxin-antitoxin (TA) systems, particularly SezAT and AbiE, within Streptococcus suis, I can evaluate the depth of the research and its contributions to our understanding of bacterial resistance mechanisms. The detailed examination of the cross-regulation between two distinct TA pairs and their influence on the stability of integrative and conjugative elements (ICEs) is noteworthy. This aspect of the study not only highlights a novel and critical mechanism but also sheds light on the broader implications for antibiotic resistance and bacterial evolution.

The authors have provided robust evidence to support their findings, demonstrating a high level of scientific rigor and innovation. Their work addresses a gap in the current literature, offering new insights that could pave the way for developing more effective strategies against antibiotic-resistant bacteria.

Given the manuscript's clear relevance to the ongoing challenges in microbiology and public health, its potential to inform future research, and its contribution to the scientific community's understanding of complex bacterial systems, I recommend its publication in PLOS pathogens. The study is well-presented, methodologically sound, and represents an advancement in the field of microbial genetics and antibiotic resistance.

In conclusion, this manuscript is not only timely but also of importance to researchers, and clinicians involved in combating antibiotic resistance. Its publication would enrich the academic discourse and inspire further research in this study area.

Thank you for your careful review and positive comments on our manuscript. We have revised the manuscript based on the valuable comments of reviewers 1 and 2, which have greatly improved the quality of the manuscript, and we hope that the current version can meet the journal’s standards for publication.
